# Combined whole-lesion radiomic and iodine analysis for differentiation of pulmonary tumors

**DOI:** 10.1038/s41598-022-15351-y

**Published:** 2022-07-12

**Authors:** Lea Azour, Jane P. Ko, Thomas O’Donnell, Nihal Patel, Priya Bhattacharji, William H. Moore

**Affiliations:** 1 Center for Biomedical Imaging, Department of Radiology, NYU Grossman School of Medicine, 660 First Avenue, New York, NY 10016 USA; 2 NYU Langone Health, New York, NY USA; 3 Siemens Healthineers, Malvern, PA USA

**Keywords:** Cancer, Medical imaging, Tomography, Computed tomography

## Abstract

Quantitative radiomic and iodine imaging features have been explored for diagnosis and characterization of tumors. In this work, we invistigate combined whole-lesion radiomic and iodine analysis for the differentiation of pulmonary tumors on contrast-enhanced dual-energy CT (DECT) chest images. 100 biopsy-proven solid lung lesions on contrast-enhanced DECT chest exams within 3 months of histopathologic sampling were identified. Lesions were volumetrically segmented using open-source software. Lesion segmentations and iodine density volumes were loaded into a radiomics prototype for quantitative analysis. Univariate analysis was performed to determine differences in volumetric iodine concentration (mean, median, maximum, minimum, 10th percentile, 90th percentile) and first and higher order radiomic features (n = 1212) between pulmonary tumors. Analyses were performed using a 2-sample t test, and filtered for false discoveries using Benjamini–Hochberg method. 100 individuals (mean age 65 ± 13 years; 59 women) with 64 primary and 36 metastatic lung lesions were included. Only one iodine concentration parameter, absolute minimum iodine, significantly differed between primary and metastatic pulmonary tumors (FDR-adjusted p = 0.015, AUC 0.69). 310 (FDR-adjusted p = 0.0008 to p = 0.0491) radiomic features differed between primary and metastatic lung tumors. Of these, 21 features achieved AUC ≥ 0.75. In subset analyses of lesions imaged by non-CTPA protocol (n = 72), 191 features significantly differed between primary and metastatic tumors, 19 of which achieved AUC ≥ 0.75. In subset analysis of tumors without history of prior treatment (n = 59), 40 features significantly differed between primary and metastatic tumors, 11 of which achieved AUC ≥ 0.75. Volumetric radiomic analysis provides differentiating capability beyond iodine quantification. While a high number of radiomic features differentiated primary versus metastatic pulmonary tumors, fewer features demonstrated good individual discriminatory utility.

## Introduction

Lung cancer is the leading cause of cancer-related death in the United States^1^ and worldwide^2^, with additional morbidity and mortality related to pulmonary metastases. CT is the most widely used imaging modality for pulmonary lesion evaluation, with serial CT or PET imaging often used to discern benign from neoplastic etiologies, and treatment response. Yet, quantitative measures, whether based on contrast enhancement or texture imaging features, have not been established to distinguish benignity from malignancy, type of neoplasm, and predict subsequent treatment response.

Contrast-enhanced dual-energy computed tomography (DECT) analysis allows iodine density to be calculated from volumes of interest^3^. Iodine concentration has been used to differentiate benign from malignant lesions and lymph nodes, including pulmonary lesions^4–7^. Iodine quantification has also been used in lung cancer for classification of tumor histopathology^6^, grade^8–10^, and treatment response^11–13^, however is affected by protocol. Because iodine values may be affected by region of interest (ROI) location and size^14^, whole-tumor three-dimensional (3D) volumetric iodine characterization^9,13^ is increasingly utilized.

Radiomics, the extraction of large-scale quantitative data from images, provides an additional method for lesion analysis. Radiomic evaluation on contrast-enhanced, including dual energy, and non-contrast enhanced studies has been used to classify tumor histopathology in non-small cell lung cancer (NSCLC)^15,16^, and predict tumor behavior and outcomes for lung cancers^10,16–22^.

To our knowledge, this is the first assessment of volumetric solid tumor characterization using DECT radiomics, which allows for the simultaneous evaluation of iodine concentration and radiomic parameters in the same cohort. Our purpose was to evaluate the performance of volumetric iodine and radiomic parameters in differentiating pulmonary tumors, including primary versus metastatic lesions.

## Materials and methods

This retrospective study was approved by the NYU institutional review board (i15-01478) and is compliant with the Health Insurance Portability and Accountability Act (HIPAA), and all methods were performed in accordance with relevant guidelines and regulations. The requirement for informed consent was waived as approved by NYU institutional review board.

A search was conducted using the radiology workflow system (Primordial, Nuance Communications Inc., Burlington, MA) for all exams acquired on our institution’s two DECT scanners (radiology search term was performing CT scanner resource) from 1/1/2015-4/30/2018. An automated follow-up search parameter required all queried radiology cases to have histopathology in the electronic medical record (Epic Systems, Verona, WI) within 90 days either before or after DECT imaging. The hospital name (appearing in all institutional pathology reports) was used as the pathology search term. Search results included patient gender, age, date of CT imaging, type of CT protocol, date of histopathology acquisition, body region imaged, histopathologic specimen, and radiology and histopathology reports.

**Figure 1:**
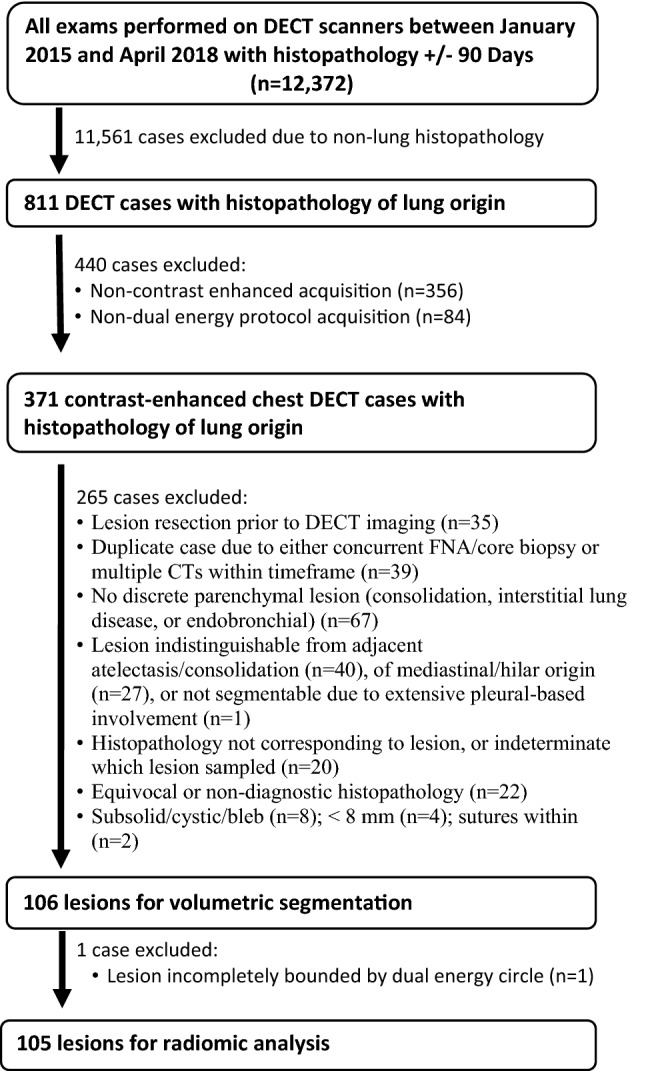
Flowchart of lesion selection and exclusion criteria. *DECT* dual energy computed tomography.

All cases were reviewed by a board-certified cardiothoracic radiologist to identify those with diagnostic lung histopathology among resulted contrast-enhanced dual-energy acquired chest CTs (Fig. 1). Exclusion criteria were lesion size < 8 mm (no size maximum); lesions indistinguishable from adjacent atelectasis/consolidation; lesions of mediastinal/hilar origin indistinguishable from adjacent central hilar vasculature; and subsolid, cystic or cavitary lesions. Lesion location was recorded. If two enhanced DECTs were resulted within the search window, the DECT temporally closest to date of histopathologic sampling was chosen. In patients with multiple lesions, only the one with definitive histopathologic sampling was chosen. Histopathology from included lesions was obtained by either core biopsy or surgical resection.

A total of 106 lesions met inclusion criteria for volumetric segmentation (Fig. 1). Collected clinical information included patient gender, age, date of tissue sampling, date of DECT imaging, lesion location, and lesion histopathology. The medical record was also reviewed for each patient to determine if any treatment for malignancy had been administered prior to image acquisition, including chemotherapy, immunotherapy, or radiotherapy. Response to therapy was not assessed. Image data were anonymized prior to analysis.

### CT imaging protocol

DECT protocol imaging was performed on a third-generation dual-source multi-detector CT (Somatom Force, Siemens Healthineers, Forchheim, Germany), with 192 × 0.6 mm collimation, tube voltages of 80 kVp (A tube) and tin filtered 150 kVp (B tube), quality reference milliampere seconds (mAs) of 130 mAs and 100 mAs, respectively, and tube current modulation (CareDose4D). The DECT data sets were reconstructed at 1 mm slice thickness at 0.8 mm increment, utilizing iterative reconstruction (ADMIRE, strength 2), with Qr40 kernel for material decomposition and dual-energy data analysis.

Contrast-enhanced chest CT (n = 74) and pulmonary CTA exams were included (n = 31). Intravenous contrast was administered using weight-based dosing of 1.5 mL/kg, for a maximum of 100 cc of 300 mg/mL of nonionic iodinated intravenous contrast, injected at a rate of 2–3 cc/s via a 20 or 22-gauge intravenous line, with a 20 s delay. For pulmonary CTA studies, 50–60 mL of nonionic iodinated intravenous contrast was administered with injection rate of 3–5 cc/s via a 20-gauge or less intravenous line, with 5–7 s delay and bolus tracking Hounsfield unit (HU) of 130 at the pulmonary trunk.

### Volumetric lesion segmentation

**Figure 2:**
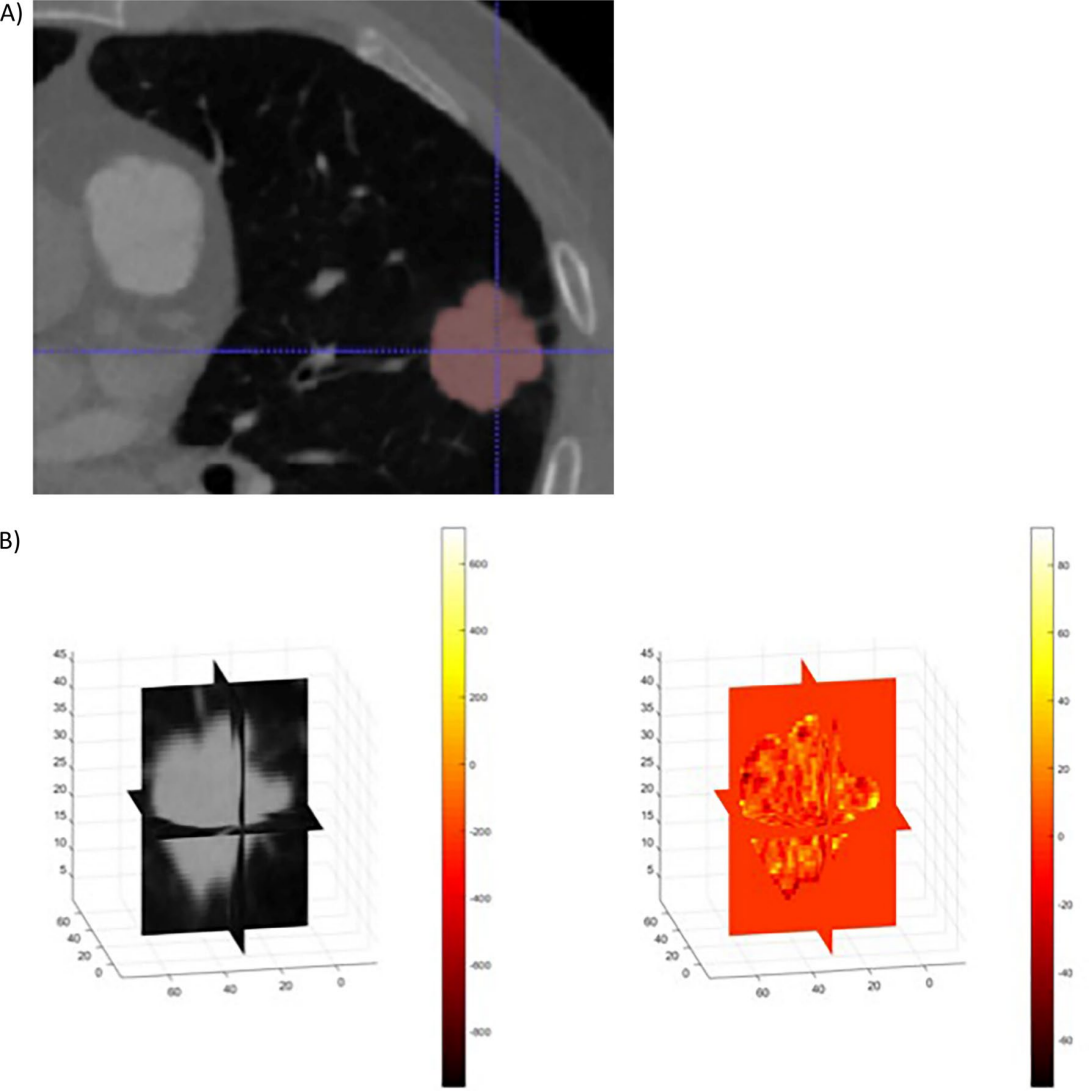
Volumetric lesion segmentation. (**A**) Axial image demonstrating lesion segmentation mask on low-kV dataset using ITK-SNAP (http://www.itksnap.org/pmwiki/pmwiki.php). (**B**) Lesion result volume reflecting iodine density (mg/mL) using R2018B Matlab 9.5.

The 106 pulmonary lesions satisfying inclusion criteria were manually volumetrically segmented on the low-kVp dataset by a cardiothoracic radiologist with one year of post-fellowship experience using an open-source software application, ITK-SNAP^23^ (Fig. 2). Segmentation retrieval failed in one case, wherein the lesion was only partially included in the dual energy circle, and this case was therefore excluded from radiomic analysis. Therefore, 105 lesions from 105 individuals (60 women, 45 men) with mean age 65 years (standard deviation 13 years, range 21–92 years) were segmented. These included primary lung cancers (n = 64) and metastatic lung tumors (n = 36) (Table 1). Benign lesions (n = 5) were excluded from iodine radiomic analysis.

**Table 1:** Lesion characteristics.

Parameter	Value
Number of lesions	100
**Lesion pathology**	All lesions (n = 100), untreated subset (n = 59)
Primary lung tumors	64, 42
Adenocarcinoma	48, 29
Squamous cell	13,12
Large cell neuroendocrine	1, 0
Sarcomatoid carcinoma	1, 1
Small cell	1, 0
Tumors metastatic to lung	36, 17
Renal/urothelial	7, 5
Colorectal	7, 2
Gynecologic	6, 2
Pancreatic	4, 0
Breast	3, 1
Melanoma	3, 3
Germ cell	1, 0
Hepatocellular	1, 1
Laryngeal squamous	1, 1
Lymphoma	1, 1
Sarcoma	1, 0
Squamous, cutaneous	1, 1
**Interval between imaging and histopathologic sampling (days)**	24 ± 24 (range − 89 to 83)
Lesions imaged before histopathologic sampling	65
Interval (days)	18 ± 19 (range 1–83)
Lesions imaged day of histopathologic sampling	2
Interval (days)	0
Lesions imaged after histopathologic sampling	33
Interval (days)	38 ± 26 (range 1–89)

The low and high kVp image data sets with the volumetrically segmented lesions were exported to a commercially available software program for DECT data (Syngo Via VB30B Dual Energy workflow, Siemens Healthineers, Forchheim, Germany). This technology allows dual energy iodine analysis, creating an “absolute” iodine density volume, comprised of voxels. This computation is applied to all voxels within the lesion volume, and thus the resultant volume has the same spatial resolution as the original low and high kVp volume pair. Processing was performed using Matlab™.

### Quantitative DECT volumetric iodine concentration and radiomic iodine texture analysis

Lesion segmentations were loaded into the investigational prototype software program for quantitative analysis (Syngo Via Frontier Radiomics, Siemens Healthineers, Forchheim, Germany). Quantitative radiomic features (n = 1212), as defined by the radiomics platform (Appendix Table 1), were extracted for each lesion. These included morphology-based shape features, first-order based iodine and histogram features, and higher order texture features based on gray level matrices.

Higher order texture matrices included gray-level co-occurrence (GLCM), gray-level size zone (GLSZM), gray-level run length (GLRLM), neighboring gray-tone difference (NGTDM) and gray-level dependence (GLDM) matrices. First and higher order texture features were also calculated from images preprocessed through filters available through the platform, using wavelet (decomposition levels: LLL, LLH, LHL, LHH, HLL, HLH, HHL, HHH), log, exponent, square, and square root functions. Each of these filters is utilized to enhance specific aspects of the underlying image for radiomic analysis. Each filter and radiomic feature pair are handled as a single feature. Shape features are intensity independent and therefore unfiltered^24^.

### Statistical analysis

Univariate analysis was performed using a 2-sample t test, filtered for false discoveries using the Benjamini–Hochberg method, to determine radiomic features significantly differing between primary versus metastatic tumors, untreated primary versus untreated metastatic tumors, lung adenocarcinoma versus squamous cell carcinomas, and untreated from treated tumors. Because exams were acquired by both CTPA and non-CTPA protocols, subset analysis of only those lesions imaged by non-CTPA protocol was also performed, for all lesions and the untreated subset. A false discovery rate (FDR) adjusted p < 0.05 significance level was used for all analyses, and features were ranked according to FDR-adjusted p < 0.05. Effect size threshold was 0.1, and decorrelation method mRMR (minimal redundancy maximal relevance). Mutual information value obtained to assess for mutual dependence of variables. Statistical analyses were performed within the Syngo Via Frontier Radiomics platform.

## Results

The analyzed cohort included 100 individuals, with 64 with primary lung cancers and 36 with metastatic lung tumors, Table 1. At time of imaging, 41 individuals had a history of prior systemic therapy (termed “treated lesions”), with one treated lesion having also undergone radiotherapy. There were 59 tumors that were treatment naïve (termed “untreated lesions”). Mean lesion volume for all lesions was 65.7 ± 138 mL. There was no significant difference in lesion volume between compared subgroups.

### Univariate analysis of radiomic features distinguishing primary versus metastatic pulmonary tumors

Of 1212 radiomic features, 310 (FDR-adjusted p = 0.0008 to p = 0.0491) significantly differed between primary and metastatic lung tumors on univariate analysis (Table 2, Appendix Table 1).

**Table 2:** Univariate analysis of radiomic features in distinguishing primary versus metastatic pulmonary tumors.

Classification task	Number of features significant at FDR-adjusted P value < .05, of 1212 total features	Corresponding range of FDR-adjusted P values	Number of significant features with AUCROC ≥ 0.75^a^	Corresponding range of AUCROC
**Primary versus metastatic tumors**				
All tumors				
All protocols (n = 100)	310	0.0008–0.0491	21	0.75–0.78
Non-CTPA exams (n = 72)	191	0.0127–0.0499	19	0.75–0.77
Untreated tumors				
All exam protocols (n = 59)	40	0.02–0.0487	11	0.75–0.81
Non-CTPA exams (n = 45)	0	–	–	–
Treated tumors, all exam protocols (n = 41)	0	–	–	–

^a^Specific features listed in Table 3.

Only one original first order iodine concentration parameter, absolute minimum iodine, significantly differed between primary and metastatic pulmonary tumors (FDR-adjusted p = 0.015; AUC 0.69). Absolute volumetric iodine minimum was higher in metastatic (− 2.4 mg/mL) than primary lung tumors (− 3.5 mg/mL). Log and squareroot functions of absolute first order minimum iodine were also significant discriminating features for primary versus metastatic lung tumors, with AUC ≥ 0.75.

In terms of shape-based features, 12 of 16 features significantly differed between primary and metastatic tumors, including compactness1 and compactness 2, major and minor axes, maximum 2D diameter (column, row and slice), spherical disproportion, sphericity, and surface volume ratio (Appendix Table 1). However, these features individually did not demonstrate AUC ≥ 0.75, and lesion volume did not significantly differ between primary and metastatic lesions.

Of the 18 first order features assessed by 12 filters (216 total parameters), 41 radiomic features significantly differed between primary and metastatic tumors. Exponential filters, computing exponential values of the original image rescaled on the range of the original image^25^, for first order features of 90th percentile, interquartile range, mean, mean absolute deviation, median, robust mean absolute deviation and root mean squared were significantly different between primary and metastatic tumors. Logarithm and square-root filtered first order entropy and uniformity significantly differed between primary and metastatic tumors. Square-root filtered absolute entropy and uniformity demonstrated AUC 0.75 (Table 3) in differentiating primary versus metastatic tumors.

**Table 3:** Significant radiomic features in distinguishing primary versus metastatic pulmonary tumors with individual AUCROC ≥ 0.75.

Primary versus metastatic lung tumors	Absolute radiomic features	AUC	MI
All tumors, all protocols (n = 100)	**squareroot_glszm_SmallAreaEmphasis**	0.78	0.13
	logarithm_glszm_SmallAreaHighGrayLevelEmphasis	0.77	0.10
	logarithm_glszm_GrayLevelVariance	0.77	0.09
	logarithm_firstorder_Minimum	0.76	0.12
	logarithm_glszm_HighGrayLevelZoneEmphasis	0.76	0.08
	logarithm_glrlm_GrayLevelVariance	0.75	0.10
	square_glszm_ZonePercentage	0.75	0.17
	squareroot_glszm_SmallAreaHighGrayLevelEmphasis	0.75	0.13
	logarithm_glszm_ZoneEntropy	0.75	0.14
	squareroot_firstorder_Uniformity	0.75	0.13
	squareroot_firstorder_Minimum	0.75	0.10
	**squareroot_glrlm_GrayLevelNonUniformityNormalized**	0.75	0.12
	squareroot_glcm_SumEntropy	0.75	0.10
	squareroot_glcm_MaximumProbability	0.75	0.15
	squareroot_glcm_JointEnergy	0.75	0.12
	logarithm_glrlm_ShortRunHighGrayLevelEmphasis	0.75	0.11
	logarithm_glrlm_GrayLevelNonUniformityNormalized	0.75	0.13
	logarithm_firstorder_Range	0.75	0.11
	logarithm_glcm_ClusterTendency	0.75	0.09
	squareroot_firstorder_Entropy	0.75	0.11
	wavelet_LHH_glszm_ZoneEntropy	0.75	0.16
All tumors, non CTPA protocol (n = 72)	square_glszm_ZonePercentage	0.77	0.20
	original_shape_Compactness2	0.77	0.16
	original_shape_Compactness1	0.77	0.21
	original_shape_Sphericity	0.77	0.21
	original_shape_SphericalDisproportion	0.77	0.18
	**squareroot_glszm_SmallAreaEmphasis**	0.77	0.15
	wavelet_LLH_glszm_SmallAreaEmphasis	0.76	0.12
	wavelet_LLH_gldm_DependenceVariance	0.76	0.20
	exponential_glszm_ZonePercentage	0.75	0.22
	squareroot_glcm_JointEnergy	0.75	0.15
	squareroot_glrlm_LongRunLowGrayLevelEmphasis	0.75	0.13
	squareroot_glszm_SmallAreaHighGrayLevelEmphasis	0.75	0.11
	**squareroot_glrlm_GrayLevelNonUniformityNormalized**	0.75	0.14
	squareroot_glcm_SumEntropy	0.75	0.12
	squareroot_gldm_LargeDependenceLowGrayLevelEmphasis	0.75	0.14
	squareroot_firstorder_Uniformity	0.75	0.15
	logarithm_firstorder_Minimum	0.75	0.11
	square_gldm_SmallDependenceEmphasis	0.75	0.14
	squareroot_firstorder_Entropy	0.75	0.11
Untreated tumors, all protocols (n = 59)	**squareroot_glszm_SmallAreaEmphasis**	0.81	0.25
	wavelet_LHH_glszm_ZoneEntropy	0.79	0.24
	original_glszm_SmallAreaEmphasis	0.79	0.16
	wavelet_LLH_glszm_SmallAreaEmphasis	0.79	0.20
	**squareroot_glrlm_GrayLevelNonUniformityNormalized**	0.77	0.17
	logarithm_glrlm_GrayLevelNonUniformityNormalized	0.76	0.12
	original_shape_Compactness2	0.76	0.13
	wavelet_LLH_glszm_ZonePercentage	0.76	0.16
	squareroot_glszm_SizeZoneNonUniformityNormalized	0.75	0.15
	wavelet_HHH_gldm_SmallDependenceLowGrayLevelEmphasis	0.75	0.11
	logarithm_glrlm_ShortRunEmphasis	0.75	0.10

*MI* mutual information value.

Of the 310 features that significantly differed between primary and metastatic lung tumors, 246 were higher order texture features. For example, several NGTDM contrast features, including original and 10 filtered (HHH, HHL, HLH, HLL, LHH, LHL, LLH, LLL, log, squareroot), significantly differed between primary and metastatic lung tumors (AUC > 0.61). NGTDM coarseness significantly differed between primary and metastatic lung tumors using 10 (log, square root, HHH, HHL, HLH, HLL, LHH, LHL, LLH, LLL) of 12 possible filters (AUC > 0.7). GLRLM run entropy significantly differed between primary and metastatic lung tumors using 9 (exponential, logarithm, square, square root, HHH, HHL, LHH, LHL, LLL) of 12 possible filters.

### Subset analysis of tumors imaged by non-CTPA protocol

In the subset of tumors imaged by non-CTPA protocol, 191 (FDR-adjusted p = 0.0127 to p = 0.0499) radiomic features differed between primary and metastatic lung tumors (Table 2).

Similar to analysis in all tumors imaged by any protocol, only one original first order iodine concentration parameter, absolute minimum iodine, significantly differed between primary and metastatic pulmonary tumors (FDR-adjusted p = 0.04; AUC 0.7). Log and squareroot functions of absolute first order minimum iodine were also significant discriminating features for primary versus metastatic lung tumors, with AUC 0.74–0.75.

In terms of shape-based features, 6 of 16 features significantly differed between primary and metastatic tumors, including compactness1, compactness 2, major axis, spherical disproportion, sphericity, and surface volume ratio. Individually, compactness1, compactness2, sphericity and spherical disproportion demonstrated AUC of 0.77. There was no significant difference in lesion volume between primary and metastatic lung tumors imaged by non-CTPA DECT protocol.

Of the 18 first order features assessed by 12 filters (216 total parameters), 33 of 216 features significantly differed between primary and metastatic tumors. Logarithm and square-root filtered entropy and uniformity were significantly different between primary and metastatic tumors, with square-root filtered first order entropy and uniformity demonstrating AUC of 0.75 (Table 3).

### Subset analysis of untreated tumors, all CT protocols

Subset analysis of untreated primary versus untreated metastatic tumors imaged by any protocol (n = 59) demonstrated significant differences in 40 radiomic features (FDR-adjusted p = 0.02 to p = 0.0487) (Table 2). Minimum iodine was not significantly different in the subset of untreated primary versus untreated metastatic tumors.

The 2 of 16 shape-based features that significantly differed between untreated primary and untreated metastatic tumors were compactness2 and surface volume ratio, the former with AUC 0.76. The remaining iodine texture features significantly differing between the untreated primary and untreated metastatic subgroups were all higher order texture features. For example, higher order features that significantly differed between the untreated primary versus untreated metastatic lung tumors included 5 of 11 NGTDM contrast features, 10 of 13 NGTDM coarseness features, and 4 of 13 GLSZM small area emphasis features, with a lesser number discriminating well based on AUC (Table 3).

### Additional classification tasks

Univariate analysis between untreated primary versus untreated metastatic tumors imaged by non-CTPA protocol (n = 45) demonstrated no significant differences in radiomic features. Univariate analysis demonstrated no significant differences in radiomic features between treated primary and treated metastatic lung tumors. There were no significant differences on univariate analysis between primary lung adenocarcinoma and squamous cell carcinomas, or treated versus untreated tumors.

## Discussion

We found radiomic analysis added discriminatory ability beyond iodine concentration. Importantly, while a high number of radiomic features significantly differed between primary and metastatic pulmonary tumors on DECT, fewer features demonstrated excellent individual performance.

We make several observations regarding the performance of radiomic features extracted from volumetrically segmented tumors on thin-section contrast-enhanced DECT exams, including comment on three main concepts: iodine concentration, shape features, and higher order radiomic features.

Original first order iodine concentration parameters were generally not significant features in differentiating between tumor histopathologies in our cohort. The benefit of volumetric analysis including iodine is the reporting of multiple “ROIs” non-reliant on subjective radiologist placement; for example, maximum, minimum, 90th and 10th percentiles are automatically derived.

Only absolute minimum iodine was significantly different between primary and metastatic lung tumors in our cohort, including in the subset of tumors imaged by non-CTPA DECT protocol. Additionally, several first order iodine-based features (mean, median, 90th percentile, interquartile range, etc.) using filters (exponential, logarithm, square, and squareroot), were significantly different between primary and metastatic tumors. Filters may potentially amplify, and thereby discern, differences based on iodine concentration. The discriminatory performance of these filtered iodine features individually reached AUC > 0.7, with reported “acceptable” performance of radiomic signatures in literature often including AUC at or above this threshold. The higher AUC threshold in our interpretation should not discount the relevance of iodine information in adding value to radiomic signatures, as features may demonstrate better performance in ensemble.

Differences in iodine by tumor type is supported by prior literature. The role of iodine quantification in predicting treatment responders has been previously demonstrated in NSCLC patients^11–13,26^. Iodine concentrations have been found to be significantly higher in epidermal growth factor receptor (EGFR)-mutated responders^11,13^, and to have a positive correlation with vascular endothelial growth factor expression^27^ and hypoxia inducible factor expression^28^ in NSCLC. Aoki et al. demonstrated primary and metastatic tumors with lower average iodine to have worse prognosis, and significantly lower rates of local control^26^. On DECT, lower iodine has been found to be associated with higher grade lung neoplasms^9^. Because iodine is associated with outcomes, and our cohort was not stratified by tumor grade or outcomes, iodine concentration parameters would not be expected to be top performers in a classification task with heterogeneity by tumor grade.

The majority of shape features were significant on univariate analysis in differentiating between primary and metastatic lesions (AUC 0.69–0.73). Notably, compactness2 (AUC 0.76) and surface to volume ratio (AUC 0.69) remained significant on univariate analysis in the subset of untreated primary versus untreated metastatic tumors, and subset of primary versus metastatic lesions imaged by non-CTPA protocol.

These two shape features have also been identified by others as reproducible^17,30,31^ and discriminating^17,31–33^. Limkin et al. showed compactness2 does not vary significantly with changes in volume, slice thickness, or resampling, and may therefore be a more reproducible radiomic feature^30^. Aerts et al., in addition to finding compactness2 to be one of the most stable radiomic features, found it to be the best performing shape feature in developing a 4-feature radiomic signature to determine prognosis in lung cancer patients^17^. Compactness2 was also shown to improve patient stratification by outcomes in another prognostic model incorporating both clinical and radiomic features in stage III NSCLC patients^32^. In a study by Shakir et al., surface to volume ratio was a stable and cancer-discriminating feature, ranking first among 105 3D features for ability to distinguish benign from malignant lung nodules^31^. In a study of subsolid nodules, surface to volume ratio was the only predictor among 92 radiomic features in differentiating benign from neoplastic nodules on univariate analysis^33^.

Our results show that many higher order texture features differentiated primary and metastatic tumors on univariate analysis, and several individually discriminated well (AUC ≥ 0.75). This supports prior research in which higher order texture features have been shown to have classifying ability, and association with clinical outcomes in oncology patients. However, higher order texture features may be less reproducible than first order or shape-based features due to influence of slice thickness or reconstruction algorithms^34^. NGTDM-derived coarseness features (reflecting the spatial rate of intensity change) were significant on univariate analyses in classifying between primary and metastatic tumors (AUC > 0.7). NGTD coarseness features have been shown to be lower in NSCLC patients who responded to treatment than non-responders, and coarseness an independent predictor of overall survival^35^. NGTDM-derived contrast features have been shown to be related to progression-free survival, with higher contrast correlating with longer progression-free survival^35^.

Higher order texture feature GLSZM small area emphasis was significant on univariate analysis in distinguishing between primary and metastatic, and untreated primary and untreated metastatic tumors with many filters. Square root filtered GLSZM small area emphasis had AUC ≥ 0.75 among all tumors all protocols, and subset analyses suggesting this feature discriminates well individually. In a phantom study of 114 texture features, GLSZM small area emphasis was identified as the factor least dependent on slice thickness^36^; feature stability may contribute to the significance of this feature across several comparisons in our study. GLSZM small area emphasis reflects underlying fine texture based on the distribution of small size zones^24^. Square-root filtered GLRLM gray level non-uniformity normalized also demonstrated good individual discriminatory utility in the overall cohort, and protocol and treatment-history based subsets.

Our study has several limitations. We did not assess the reproducibility of radiomic features, which is variable^37,38^. Future study in larger cohorts may determine optimal methods for dimensionality reduction based on statistical methods, including intra-and inter-observer variability^39^, and/or machine learning. Though Aerts et al. has noted that more stable features generally demonstrate better performance^17^. Our subgroups were relatively small, precluding meaningful multivariable analysis with validation and test experiments. We included some pulmonary angiograms, however performed subset analyses of lesions imaged by non-CTPA DECT protocol. Standardization of imaging features–normalization in terms of not only contrast but also noise and intensity level for example, has been suggested to improve radiomic-based histopathologic prediction^29^, and may also be a necessary step when incorporating exams obtained by various contrast-enhanced protocols, scanners, or institutions, and when assessing delta radiomics on contrast-enhanced studies. Effective radiomic signatures should be robust across imaging techniques to be clinically practical. We did not investigate specific treatment effects, and provided subgroup analyses excluding the potentially heterogeneously treated lesions. Evaluation of the peri-tumoral region, shown to improve lesion classification^40^, and incorporation of tumor and patient characteristics may also strengthen predictive analyses in future modeling.

In conclusion, DECT radiomic features allow discriminatory potential beyond that of iodine concentration. Identifying radiomic features that independently discriminate well may direct understanding and development of reliable ensemble radiomic signatures.

## Supplementary Information

Supplementary Information.

## Data Availability

The datasets used and/or analyzed during the current study are available from the corresponding author on reasonable request.
