# Supplementary material for: Combined whole-lesion radiomic and iodine analysis for differentiation of pulmonary tumors
Source: Sci Rep. 2022 Jul 12;12:11813. doi: 10.1038/s41598-022-15351-y (PMC9276812; doi:10.1038/s41598-022-15351-y)
Supplement: Supplementary file 1 — Supplementary Information. [file 41598_2022_15351_MOESM1_ESM.docx]

**Supplementary Table 1: Texture features significantly differing between primary and metastatic tumors on univariate analysis**

| **Primary v. Metastatic Lung Tumors,**  **all DECT protocols** | **Untreated Primary v. Untreated Metastatic Lung Tumors,**  **all DECT protocols** |
| --- | --- |
| **Absolute** | **Absolute** |
| **original_shape_Compactness1** | original_shape_Compactness1 |
| **original_shape_Compactness2** | **original_shape_Compactness2** |
| original_shape_Elongation | original_shape_Elongation |
| original_shape_Flatness | original_shape_Flatness |
| **original_shape_LeastAxis** | original_shape_LeastAxis |
| **original_shape_MajorAxis** | original_shape_MajorAxis |
| **original_shape_Maximum2DDiameterColumn** | original_shape_Maximum2DDiameterColumn |
| **original_shape_Maximum2DDiameterRow** | original_shape_Maximum2DDiameterRow |
| **original_shape_Maximum2DDiameterSlice** | original_shape_Maximum2DDiameterSlice |
| **original_shape_Maximum3DDiameter** | original_shape_Maximum3DDiameter |
| **original_shape_MinorAxis** | original_shape_MinorAxis |
| **original_shape_SphericalDisproportion** | original_shape_SphericalDisproportion |
| **original_shape_Sphericity** | original_shape_Sphericity |
| original_shape_SurfaceArea | original_shape_SurfaceArea |
| **original_shape_SurfaceVolumeRatio** | **original_shape_SurfaceVolumeRatio** |
| original_shape_Volume | original_shape_Volume |
| original_firstorder_10Percentile | original_firstorder_10Percentile |
| original_firstorder_90Percentile | original_firstorder_90Percentile |
| original_firstorder_Energy | original_firstorder_Energy |
| original_firstorder_Entropy | original_firstorder_Entropy |
| original_firstorder_InterquartileRange | original_firstorder_InterquartileRange |
| original_firstorder_Kurtosis | original_firstorder_Kurtosis |
| original_firstorder_Maximum | original_firstorder_Maximum |
| original_firstorder_Mean | original_firstorder_Mean |
| original_firstorder_MeanAbsoluteDeviation | original_firstorder_MeanAbsoluteDeviation |
| original_firstorder_Median | original_firstorder_Median |
| **original_firstorder_Minimum** | original_firstorder_Minimum |
| original_firstorder_Range | original_firstorder_Range |
| original_firstorder_RobustMeanAbsoluteDeviation | original_firstorder_RobustMeanAbsoluteDeviation |
| original_firstorder_RootMeanSquared | original_firstorder_RootMeanSquared |
| original_firstorder_Skewness | original_firstorder_Skewness |
| original_firstorder_TotalEnergy | original_firstorder_TotalEnergy |
| original_firstorder_Uniformity | original_firstorder_Uniformity |
| original_firstorder_Variance | original_firstorder_Variance |
| exponential_firstorder_10Percentile | exponential_firstorder_10Percentile |
| **exponential_firstorder_90Percentile** | exponential_firstorder_90Percentile |
| exponential_firstorder_Energy | exponential_firstorder_Energy |
| exponential_firstorder_Entropy | exponential_firstorder_Entropy |
| **exponential_firstorder_InterquartileRange** | exponential_firstorder_InterquartileRange |
| exponential_firstorder_Kurtosis | exponential_firstorder_Kurtosis |
| exponential_firstorder_Maximum | exponential_firstorder_Maximum |
| **exponential_firstorder_Mean** | exponential_firstorder_Mean |
| **exponential_firstorder_MeanAbsoluteDeviation** | exponential_firstorder_MeanAbsoluteDeviation |
| **exponential_firstorder_Median** | exponential_firstorder_Median |
| exponential_firstorder_Minimum | exponential_firstorder_Minimum |
| exponential_firstorder_Range | exponential_firstorder_Range |
| **exponential_firstorder_RobustMeanAbsoluteDeviation** | exponential_firstorder_RobustMeanAbsoluteDeviation |
| **exponential_firstorder_RootMeanSquared** | exponential_firstorder_RootMeanSquared |
| exponential_firstorder_Skewness | exponential_firstorder_Skewness |
| exponential_firstorder_TotalEnergy | exponential_firstorder_TotalEnergy |
| exponential_firstorder_Uniformity | exponential_firstorder_Uniformity |
| exponential_firstorder_Variance | exponential_firstorder_Variance |
| logarithm_firstorder_10Percentile | logarithm_firstorder_10Percentile |
| **logarithm_firstorder_90Percentile** | logarithm_firstorder_90Percentile |
| logarithm_firstorder_Energy | logarithm_firstorder_Energy |
| **logarithm_firstorder_Entropy** | logarithm_firstorder_Entropy |
| **logarithm_firstorder_InterquartileRange** | logarithm_firstorder_InterquartileRange |
| logarithm_firstorder_Kurtosis | logarithm_firstorder_Kurtosis |
| logarithm_firstorder_Maximum | logarithm_firstorder_Maximum |
| logarithm_firstorder_Mean | logarithm_firstorder_Mean |
| **logarithm_firstorder_MeanAbsoluteDeviation** | logarithm_firstorder_MeanAbsoluteDeviation |
| **logarithm_firstorder_Median** | logarithm_firstorder_Median |
| **logarithm_firstorder_Minimum** | logarithm_firstorder_Minimum |
| **logarithm_firstorder_Range** | logarithm_firstorder_Range |
| **logarithm_firstorder_RobustMeanAbsoluteDeviation** | logarithm_firstorder_RobustMeanAbsoluteDeviation |
| **logarithm_firstorder_RootMeanSquared** | logarithm_firstorder_RootMeanSquared |
| logarithm_firstorder_Skewness | logarithm_firstorder_Skewness |
| logarithm_firstorder_TotalEnergy | logarithm_firstorder_TotalEnergy |
| **logarithm_firstorder_Uniformity** | logarithm_firstorder_Uniformity |
| logarithm_firstorder_Variance | logarithm_firstorder_Variance |
| square_firstorder_10Percentile | square_firstorder_10Percentile |
| **square_firstorder_90Percentile** | square_firstorder_90Percentile |
| square_firstorder_Energy | square_firstorder_Energy |
| square_firstorder_Entropy | square_firstorder_Entropy |
| **square_firstorder_InterquartileRange** | square_firstorder_InterquartileRange |
| square_firstorder_Kurtosis | square_firstorder_Kurtosis |
| square_firstorder_Maximum | square_firstorder_Maximum |
| **square_firstorder_Mean** | square_firstorder_Mean |
| **square_firstorder_MeanAbsoluteDeviation** | square_firstorder_MeanAbsoluteDeviation |
| **square_firstorder_Median** | square_firstorder_Median |
| square_firstorder_Minimum | square_firstorder_Minimum |
| square_firstorder_Range | square_firstorder_Range |
| **square_firstorder_RobustMeanAbsoluteDeviation** | square_firstorder_RobustMeanAbsoluteDeviation |
| **square_firstorder_RootMeanSquared** | square_firstorder_RootMeanSquared |
| square_firstorder_Skewness | square_firstorder_Skewness |
| square_firstorder_TotalEnergy | square_firstorder_TotalEnergy |
| square_firstorder_Uniformity | square_firstorder_Uniformity |
| **square_firstorder_Variance** | square_firstorder_Variance |
| squareroot_firstorder_10Percentile | squareroot_firstorder_10Percentile |
| **squareroot_firstorder_90Percentile** | squareroot_firstorder_90Percentile |
| squareroot_firstorder_Energy | squareroot_firstorder_Energy |
| **squareroot_firstorder_Entropy** | squareroot_firstorder_Entropy |
| **squareroot_firstorder_InterquartileRange** | squareroot_firstorder_InterquartileRange |
| squareroot_firstorder_Kurtosis | squareroot_firstorder_Kurtosis |
| **squareroot_firstorder_Maximum** | squareroot_firstorder_Maximum |
| squareroot_firstorder_Mean | squareroot_firstorder_Mean |
| **squareroot_firstorder_MeanAbsoluteDeviation** | squareroot_firstorder_MeanAbsoluteDeviation |
| squareroot_firstorder_Median | squareroot_firstorder_Median |
| **squareroot_firstorder_Minimum** | squareroot_firstorder_Minimum |
| **squareroot_firstorder_Range** | squareroot_firstorder_Range |
| **squareroot_firstorder_RobustMeanAbsoluteDeviation** | squareroot_firstorder_RobustMeanAbsoluteDeviation |
| **squareroot_firstorder_RootMeanSquared** | squareroot_firstorder_RootMeanSquared |
| squareroot_firstorder_Skewness | squareroot_firstorder_Skewness |
| squareroot_firstorder_TotalEnergy | squareroot_firstorder_TotalEnergy |
| **squareroot_firstorder_Uniformity** | squareroot_firstorder_Uniformity |
| **squareroot_firstorder_Variance** | squareroot_firstorder_Variance |
| wavelet_HHH_firstorder_10Percentile | wavelet_HHH_firstorder_10Percentile |
| wavelet_HHH_firstorder_90Percentile | wavelet_HHH_firstorder_90Percentile |
| wavelet_HHH_firstorder_Energy | wavelet_HHH_firstorder_Energy |
| wavelet_HHH_firstorder_Entropy | wavelet_HHH_firstorder_Entropy |
| wavelet_HHH_firstorder_InterquartileRange | wavelet_HHH_firstorder_InterquartileRange |
| wavelet_HHH_firstorder_Kurtosis | wavelet_HHH_firstorder_Kurtosis |
| wavelet_HHH_firstorder_Maximum | wavelet_HHH_firstorder_Maximum |
| wavelet_HHH_firstorder_Mean | wavelet_HHH_firstorder_Mean |
| wavelet_HHH_firstorder_MeanAbsoluteDeviation | wavelet_HHH_firstorder_MeanAbsoluteDeviation |
| wavelet_HHH_firstorder_Median | wavelet_HHH_firstorder_Median |
| wavelet_HHH_firstorder_Minimum | wavelet_HHH_firstorder_Minimum |
| wavelet_HHH_firstorder_Range | wavelet_HHH_firstorder_Range |
| wavelet_HHH_firstorder_RobustMeanAbsoluteDeviation | wavelet_HHH_firstorder_RobustMeanAbsoluteDeviation |
| wavelet_HHH_firstorder_RootMeanSquared | wavelet_HHH_firstorder_RootMeanSquared |
| wavelet_HHH_firstorder_Skewness | wavelet_HHH_firstorder_Skewness |
| wavelet_HHH_firstorder_TotalEnergy | wavelet_HHH_firstorder_TotalEnergy |
| wavelet_HHH_firstorder_Uniformity | wavelet_HHH_firstorder_Uniformity |
| wavelet_HHH_firstorder_Variance | wavelet_HHH_firstorder_Variance |
| wavelet_HHL_firstorder_10Percentile | wavelet_HHL_firstorder_10Percentile |
| wavelet_HHL_firstorder_90Percentile | wavelet_HHL_firstorder_90Percentile |
| wavelet_HHL_firstorder_Energy | wavelet_HHL_firstorder_Energy |
| wavelet_HHL_firstorder_Entropy | wavelet_HHL_firstorder_Entropy |
| wavelet_HHL_firstorder_InterquartileRange | wavelet_HHL_firstorder_InterquartileRange |
| wavelet_HHL_firstorder_Kurtosis | wavelet_HHL_firstorder_Kurtosis |
| **wavelet_HHL_firstorder_Maximum** | wavelet_HHL_firstorder_Maximum |
| wavelet_HHL_firstorder_Mean | wavelet_HHL_firstorder_Mean |
| wavelet_HHL_firstorder_MeanAbsoluteDeviation | wavelet_HHL_firstorder_MeanAbsoluteDeviation |
| wavelet_HHL_firstorder_Median | wavelet_HHL_firstorder_Median |
| wavelet_HHL_firstorder_Minimum | wavelet_HHL_firstorder_Minimum |
| **wavelet_HHL_firstorder_Range** | wavelet_HHL_firstorder_Range |
| wavelet_HHL_firstorder_RobustMeanAbsoluteDeviation | wavelet_HHL_firstorder_RobustMeanAbsoluteDeviation |
| wavelet_HHL_firstorder_RootMeanSquared | wavelet_HHL_firstorder_RootMeanSquared |
| wavelet_HHL_firstorder_Skewness | wavelet_HHL_firstorder_Skewness |
| wavelet_HHL_firstorder_TotalEnergy | wavelet_HHL_firstorder_TotalEnergy |
| wavelet_HHL_firstorder_Uniformity | wavelet_HHL_firstorder_Uniformity |
| wavelet_HHL_firstorder_Variance | wavelet_HHL_firstorder_Variance |
| wavelet_HLH_firstorder_10Percentile | wavelet_HLH_firstorder_10Percentile |
| wavelet_HLH_firstorder_90Percentile | wavelet_HLH_firstorder_90Percentile |
| wavelet_HLH_firstorder_Energy | wavelet_HLH_firstorder_Energy |
| wavelet_HLH_firstorder_Entropy | wavelet_HLH_firstorder_Entropy |
| wavelet_HLH_firstorder_InterquartileRange | wavelet_HLH_firstorder_InterquartileRange |
| wavelet_HLH_firstorder_Kurtosis | wavelet_HLH_firstorder_Kurtosis |
| wavelet_HLH_firstorder_Maximum | wavelet_HLH_firstorder_Maximum |
| wavelet_HLH_firstorder_Mean | wavelet_HLH_firstorder_Mean |
| wavelet_HLH_firstorder_MeanAbsoluteDeviation | wavelet_HLH_firstorder_MeanAbsoluteDeviation |
| wavelet_HLH_firstorder_Median | wavelet_HLH_firstorder_Median |
| wavelet_HLH_firstorder_Minimum | wavelet_HLH_firstorder_Minimum |
| wavelet_HLH_firstorder_Range | wavelet_HLH_firstorder_Range |
| wavelet_HLH_firstorder_RobustMeanAbsoluteDeviation | wavelet_HLH_firstorder_RobustMeanAbsoluteDeviation |
| wavelet_HLH_firstorder_RootMeanSquared | wavelet_HLH_firstorder_RootMeanSquared |
| wavelet_HLH_firstorder_Skewness | wavelet_HLH_firstorder_Skewness |
| wavelet_HLH_firstorder_TotalEnergy | wavelet_HLH_firstorder_TotalEnergy |
| wavelet_HLH_firstorder_Uniformity | wavelet_HLH_firstorder_Uniformity |
| wavelet_HLH_firstorder_Variance | wavelet_HLH_firstorder_Variance |
| wavelet_HLL_firstorder_10Percentile | wavelet_HLL_firstorder_10Percentile |
| wavelet_HLL_firstorder_90Percentile | wavelet_HLL_firstorder_90Percentile |
| wavelet_HLL_firstorder_Energy | wavelet_HLL_firstorder_Energy |
| wavelet_HLL_firstorder_Entropy | wavelet_HLL_firstorder_Entropy |
| wavelet_HLL_firstorder_InterquartileRange | wavelet_HLL_firstorder_InterquartileRange |
| wavelet_HLL_firstorder_Kurtosis | wavelet_HLL_firstorder_Kurtosis |
| wavelet_HLL_firstorder_Maximum | wavelet_HLL_firstorder_Maximum |
| wavelet_HLL_firstorder_Mean | wavelet_HLL_firstorder_Mean |
| wavelet_HLL_firstorder_MeanAbsoluteDeviation | wavelet_HLL_firstorder_MeanAbsoluteDeviation |
| wavelet_HLL_firstorder_Median | wavelet_HLL_firstorder_Median |
| wavelet_HLL_firstorder_Minimum | wavelet_HLL_firstorder_Minimum |
| wavelet_HLL_firstorder_Range | wavelet_HLL_firstorder_Range |
| wavelet_HLL_firstorder_RobustMeanAbsoluteDeviation | wavelet_HLL_firstorder_RobustMeanAbsoluteDeviation |
| wavelet_HLL_firstorder_RootMeanSquared | wavelet_HLL_firstorder_RootMeanSquared |
| wavelet_HLL_firstorder_Skewness | wavelet_HLL_firstorder_Skewness |
| wavelet_HLL_firstorder_TotalEnergy | wavelet_HLL_firstorder_TotalEnergy |
| wavelet_HLL_firstorder_Uniformity | wavelet_HLL_firstorder_Uniformity |
| wavelet_HLL_firstorder_Variance | wavelet_HLL_firstorder_Variance |
| wavelet_LHH_firstorder_10Percentile | wavelet_LHH_firstorder_10Percentile |
| wavelet_LHH_firstorder_90Percentile | wavelet_LHH_firstorder_90Percentile |
| wavelet_LHH_firstorder_Energy | wavelet_LHH_firstorder_Energy |
| wavelet_LHH_firstorder_Entropy | wavelet_LHH_firstorder_Entropy |
| wavelet_LHH_firstorder_InterquartileRange | wavelet_LHH_firstorder_InterquartileRange |
| wavelet_LHH_firstorder_Kurtosis | wavelet_LHH_firstorder_Kurtosis |
| wavelet_LHH_firstorder_Maximum | wavelet_LHH_firstorder_Maximum |
| wavelet_LHH_firstorder_Mean | wavelet_LHH_firstorder_Mean |
| wavelet_LHH_firstorder_MeanAbsoluteDeviation | wavelet_LHH_firstorder_MeanAbsoluteDeviation |
| wavelet_LHH_firstorder_Median | wavelet_LHH_firstorder_Median |
| wavelet_LHH_firstorder_Minimum | wavelet_LHH_firstorder_Minimum |
| **wavelet_LHH_firstorder_Range** | wavelet_LHH_firstorder_Range |
| wavelet_LHH_firstorder_RobustMeanAbsoluteDeviation | wavelet_LHH_firstorder_RobustMeanAbsoluteDeviation |
| wavelet_LHH_firstorder_RootMeanSquared | wavelet_LHH_firstorder_RootMeanSquared |
| wavelet_LHH_firstorder_Skewness | wavelet_LHH_firstorder_Skewness |
| wavelet_LHH_firstorder_TotalEnergy | wavelet_LHH_firstorder_TotalEnergy |
| wavelet_LHH_firstorder_Uniformity | wavelet_LHH_firstorder_Uniformity |
| wavelet_LHH_firstorder_Variance | wavelet_LHH_firstorder_Variance |
| wavelet_LLH_firstorder_10Percentile | wavelet_LLH_firstorder_10Percentile |
| wavelet_LLH_firstorder_90Percentile | wavelet_LLH_firstorder_90Percentile |
| wavelet_LLH_firstorder_Energy | wavelet_LLH_firstorder_Energy |
| wavelet_LLH_firstorder_Entropy | wavelet_LLH_firstorder_Entropy |
| wavelet_LLH_firstorder_InterquartileRange | wavelet_LLH_firstorder_InterquartileRange |
| wavelet_LLH_firstorder_Kurtosis | wavelet_LLH_firstorder_Kurtosis |
| wavelet_LLH_firstorder_Maximum | wavelet_LLH_firstorder_Maximum |
| wavelet_LLH_firstorder_Mean | wavelet_LLH_firstorder_Mean |
| wavelet_LLH_firstorder_MeanAbsoluteDeviation | wavelet_LLH_firstorder_MeanAbsoluteDeviation |
| wavelet_LLH_firstorder_Median | wavelet_LLH_firstorder_Median |
| wavelet_LLH_firstorder_Minimum | wavelet_LLH_firstorder_Minimum |
| wavelet_LLH_firstorder_Range | wavelet_LLH_firstorder_Range |
| wavelet_LLH_firstorder_RobustMeanAbsoluteDeviation | wavelet_LLH_firstorder_RobustMeanAbsoluteDeviation |
| wavelet_LLH_firstorder_RootMeanSquared | wavelet_LLH_firstorder_RootMeanSquared |
| wavelet_LLH_firstorder_Skewness | wavelet_LLH_firstorder_Skewness |
| wavelet_LLH_firstorder_TotalEnergy | wavelet_LLH_firstorder_TotalEnergy |
| wavelet_LLH_firstorder_Uniformity | wavelet_LLH_firstorder_Uniformity |
| wavelet_LLH_firstorder_Variance | wavelet_LLH_firstorder_Variance |
| wavelet_LHL_firstorder_10Percentile | wavelet_LHL_firstorder_10Percentile |
| wavelet_LHL_firstorder_90Percentile | wavelet_LHL_firstorder_90Percentile |
| wavelet_LHL_firstorder_Energy | wavelet_LHL_firstorder_Energy |
| wavelet_LHL_firstorder_Entropy | wavelet_LHL_firstorder_Entropy |
| wavelet_LHL_firstorder_InterquartileRange | wavelet_LHL_firstorder_InterquartileRange |
| wavelet_LHL_firstorder_Kurtosis | wavelet_LHL_firstorder_Kurtosis |
| **wavelet_LHL_firstorder_Maximum** | wavelet_LHL_firstorder_Maximum |
| wavelet_LHL_firstorder_Mean | wavelet_LHL_firstorder_Mean |
| wavelet_LHL_firstorder_MeanAbsoluteDeviation | wavelet_LHL_firstorder_MeanAbsoluteDeviation |
| wavelet_LHL_firstorder_Median | wavelet_LHL_firstorder_Median |
| wavelet_LHL_firstorder_Minimum | wavelet_LHL_firstorder_Minimum |
| wavelet_LHL_firstorder_Range | wavelet_LHL_firstorder_Range |
| wavelet_LHL_firstorder_RobustMeanAbsoluteDeviation | wavelet_LHL_firstorder_RobustMeanAbsoluteDeviation |
| wavelet_LHL_firstorder_RootMeanSquared | wavelet_LHL_firstorder_RootMeanSquared |
| wavelet_LHL_firstorder_Skewness | wavelet_LHL_firstorder_Skewness |
| wavelet_LHL_firstorder_TotalEnergy | wavelet_LHL_firstorder_TotalEnergy |
| wavelet_LHL_firstorder_Uniformity | wavelet_LHL_firstorder_Uniformity |
| wavelet_LHL_firstorder_Variance | wavelet_LHL_firstorder_Variance |
| wavelet_LLL_firstorder_10Percentile | wavelet_LLL_firstorder_10Percentile |
| wavelet_LLL_firstorder_90Percentile | wavelet_LLL_firstorder_90Percentile |
| wavelet_LLL_firstorder_Energy | wavelet_LLL_firstorder_Energy |
| wavelet_LLL_firstorder_Entropy | wavelet_LLL_firstorder_Entropy |
| wavelet_LLL_firstorder_InterquartileRange | wavelet_LLL_firstorder_InterquartileRange |
| wavelet_LLL_firstorder_Kurtosis | wavelet_LLL_firstorder_Kurtosis |
| wavelet_LLL_firstorder_Maximum | wavelet_LLL_firstorder_Maximum |
| wavelet_LLL_firstorder_Mean | wavelet_LLL_firstorder_Mean |
| wavelet_LLL_firstorder_MeanAbsoluteDeviation | wavelet_LLL_firstorder_MeanAbsoluteDeviation |
| wavelet_LLL_firstorder_Median | wavelet_LLL_firstorder_Median |
| wavelet_LLL_firstorder_Minimum | wavelet_LLL_firstorder_Minimum |
| wavelet_LLL_firstorder_Range | wavelet_LLL_firstorder_Range |
| wavelet_LLL_firstorder_RobustMeanAbsoluteDeviation | wavelet_LLL_firstorder_RobustMeanAbsoluteDeviation |
| wavelet_LLL_firstorder_RootMeanSquared | wavelet_LLL_firstorder_RootMeanSquared |
| wavelet_LLL_firstorder_Skewness | wavelet_LLL_firstorder_Skewness |
| wavelet_LLL_firstorder_TotalEnergy | wavelet_LLL_firstorder_TotalEnergy |
| wavelet_LLL_firstorder_Uniformity | wavelet_LLL_firstorder_Uniformity |
| wavelet_LLL_firstorder_Variance | wavelet_LLL_firstorder_Variance |
| exponential_glcm_Autocorrelation | exponential_glcm_Autocorrelation |
| exponential_glcm_ClusterProminence | exponential_glcm_ClusterProminence |
| exponential_glcm_ClusterShade | exponential_glcm_ClusterShade |
| exponential_glcm_ClusterTendency | exponential_glcm_ClusterTendency |
| exponential_glcm_Contrast | exponential_glcm_Contrast |
| exponential_glcm_Correlation | exponential_glcm_Correlation |
| exponential_glcm_DifferenceAverage | exponential_glcm_DifferenceAverage |
| exponential_glcm_DifferenceEntropy | exponential_glcm_DifferenceEntropy |
| exponential_glcm_DifferenceVariance | exponential_glcm_DifferenceVariance |
| exponential_glcm_Id | exponential_glcm_Id |
| exponential_glcm_Idm | exponential_glcm_Idm |
| exponential_glcm_Idmn | exponential_glcm_Idmn |
| exponential_glcm_Idn | exponential_glcm_Idn |
| exponential_glcm_Imc1 | exponential_glcm_Imc1 |
| exponential_glcm_Imc2 | exponential_glcm_Imc2 |
| exponential_glcm_InverseVariance | exponential_glcm_InverseVariance |
| exponential_glcm_JointAverage | exponential_glcm_JointAverage |
| exponential_glcm_JointEnergy | exponential_glcm_JointEnergy |
| exponential_glcm_JointEntropy | exponential_glcm_JointEntropy |
| exponential_glcm_MaximumProbability | exponential_glcm_MaximumProbability |
| exponential_glcm_SumAverage | exponential_glcm_SumAverage |
| exponential_glcm_SumEntropy | exponential_glcm_SumEntropy |
| exponential_glcm_SumSquares | exponential_glcm_SumSquares |
| **exponential_gldm_DependenceEntropy** | exponential_gldm_DependenceEntropy |
| exponential_gldm_DependenceNonUniformity | exponential_gldm_DependenceNonUniformity |
| **exponential_gldm_DependenceNonUniformityNormalized** | exponential_gldm_DependenceNonUniformityNormalized |
| **exponential_gldm_DependenceVariance** | exponential_gldm_DependenceVariance |
| exponential_gldm_GrayLevelNonUniformity | exponential_gldm_GrayLevelNonUniformity |
| exponential_gldm_GrayLevelVariance | exponential_gldm_GrayLevelVariance |
| exponential_gldm_HighGrayLevelEmphasis | exponential_gldm_HighGrayLevelEmphasis |
| **exponential_gldm_LargeDependenceEmphasis** | **exponential_gldm_LargeDependenceEmphasis** |
| **exponential_gldm_LargeDependenceHighGrayLevelEmphasis** | **exponential_gldm_LargeDependenceHighGrayLevelEmphasis** |
| **exponential_gldm_LargeDependenceLowGrayLevelEmphasis** | **exponential_gldm_LargeDependenceLowGrayLevelEmphasis** |
| exponential_gldm_LowGrayLevelEmphasis | exponential_gldm_LowGrayLevelEmphasis |
| **exponential_gldm_SmallDependenceEmphasis** | exponential_gldm_SmallDependenceEmphasis |
| **exponential_gldm_SmallDependenceHighGrayLevelEmphasis** | exponential_gldm_SmallDependenceHighGrayLevelEmphasis |
| **exponential_gldm_SmallDependenceLowGrayLevelEmphasis** | **exponential_gldm_SmallDependenceLowGrayLevelEmphasis** |
| exponential_glrlm_GrayLevelNonUniformity | exponential_glrlm_GrayLevelNonUniformity |
| exponential_glrlm_GrayLevelNonUniformityNormalized | exponential_glrlm_GrayLevelNonUniformityNormalized |
| exponential_glrlm_GrayLevelVariance | exponential_glrlm_GrayLevelVariance |
| exponential_glrlm_HighGrayLevelRunEmphasis | exponential_glrlm_HighGrayLevelRunEmphasis |
| exponential_glrlm_LongRunEmphasis | exponential_glrlm_LongRunEmphasis |
| exponential_glrlm_LongRunHighGrayLevelEmphasis | exponential_glrlm_LongRunHighGrayLevelEmphasis |
| exponential_glrlm_LongRunLowGrayLevelEmphasis | exponential_glrlm_LongRunLowGrayLevelEmphasis |
| exponential_glrlm_LowGrayLevelRunEmphasis | exponential_glrlm_LowGrayLevelRunEmphasis |
| **exponential_glrlm_RunEntropy** | exponential_glrlm_RunEntropy |
| exponential_glrlm_RunLengthNonUniformity | exponential_glrlm_RunLengthNonUniformity |
| **exponential_glrlm_RunLengthNonUniformityNormalized** | **exponential_glrlm_RunLengthNonUniformityNormalized** |
| **exponential_glrlm_RunPercentage** | **exponential_glrlm_RunPercentage** |
| exponential_glrlm_RunVariance | exponential_glrlm_RunVariance |
| **exponential_glrlm_ShortRunEmphasis** | exponential_glrlm_ShortRunEmphasis |
| **exponential_glrlm_ShortRunHighGrayLevelEmphasis** | exponential_glrlm_ShortRunHighGrayLevelEmphasis |
| **exponential_glrlm_ShortRunLowGrayLevelEmphasis** | exponential_glrlm_ShortRunLowGrayLevelEmphasis |
| exponential_glszm_GrayLevelNonUniformity | exponential_glszm_GrayLevelNonUniformity |
| exponential_glszm_GrayLevelNonUniformityNormalized | exponential_glszm_GrayLevelNonUniformityNormalized |
| exponential_glszm_GrayLevelVariance | exponential_glszm_GrayLevelVariance |
| exponential_glszm_HighGrayLevelZoneEmphasis | exponential_glszm_HighGrayLevelZoneEmphasis |
| exponential_glszm_LargeAreaEmphasis | exponential_glszm_LargeAreaEmphasis |
| exponential_glszm_LargeAreaHighGrayLevelEmphasis | exponential_glszm_LargeAreaHighGrayLevelEmphasis |
| exponential_glszm_LargeAreaLowGrayLevelEmphasis | exponential_glszm_LargeAreaLowGrayLevelEmphasis |
| exponential_glszm_LowGrayLevelZoneEmphasis | exponential_glszm_LowGrayLevelZoneEmphasis |
| exponential_glszm_SizeZoneNonUniformity | exponential_glszm_SizeZoneNonUniformity |
| exponential_glszm_SizeZoneNonUniformityNormalized | exponential_glszm_SizeZoneNonUniformityNormalized |
| exponential_glszm_SmallAreaEmphasis | exponential_glszm_SmallAreaEmphasis |
| exponential_glszm_SmallAreaHighGrayLevelEmphasis | exponential_glszm_SmallAreaHighGrayLevelEmphasis |
| exponential_glszm_SmallAreaLowGrayLevelEmphasis | exponential_glszm_SmallAreaLowGrayLevelEmphasis |
| exponential_glszm_ZoneEntropy | exponential_glszm_ZoneEntropy |
| **exponential_glszm_ZonePercentage** | exponential_glszm_ZonePercentage |
| exponential_glszm_ZoneVariance | exponential_glszm_ZoneVariance |
| exponential_ngtdm_Busyness | exponential_ngtdm_Busyness |
| exponential_ngtdm_Coarseness | exponential_ngtdm_Coarseness |
| exponential_ngtdm_Complexity | exponential_ngtdm_Complexity |
| exponential_ngtdm_Strength | exponential_ngtdm_Strength |
| **logarithm_glcm_Autocorrelation** | logarithm_glcm_Autocorrelation |
| logarithm_glcm_ClusterProminence | logarithm_glcm_ClusterProminence |
| logarithm_glcm_ClusterShade | logarithm_glcm_ClusterShade |
| **logarithm_glcm_ClusterTendency** | logarithm_glcm_ClusterTendency |
| logarithm_glcm_Contrast | logarithm_glcm_Contrast |
| logarithm_glcm_Correlation | logarithm_glcm_Correlation |
| **logarithm_glcm_DifferenceAverage** | logarithm_glcm_DifferenceAverage |
| **logarithm_glcm_DifferenceEntropy** | logarithm_glcm_DifferenceEntropy |
| logarithm_glcm_DifferenceVariance | logarithm_glcm_DifferenceVariance |
| **logarithm_glcm_Id** | logarithm_glcm_Id |
| **logarithm_glcm_Idm** | logarithm_glcm_Idm |
| logarithm_glcm_Idmn | logarithm_glcm_Idmn |
| logarithm_glcm_Idn | logarithm_glcm_Idn |
| logarithm_glcm_Imc1 | logarithm_glcm_Imc1 |
| **logarithm_glcm_Imc2** | logarithm_glcm_Imc2 |
| logarithm_glcm_InverseVariance | logarithm_glcm_InverseVariance |
| **logarithm_glcm_JointAverage** | logarithm_glcm_JointAverage |
| **logarithm_glcm_JointEnergy** | logarithm_glcm_JointEnergy |
| **logarithm_glcm_JointEntropy** | logarithm_glcm_JointEntropy |
| **logarithm_glcm_MaximumProbability** | logarithm_glcm_MaximumProbability |
| **logarithm_glcm_SumAverage** | logarithm_glcm_SumAverage |
| **logarithm_glcm_SumEntropy** | logarithm_glcm_SumEntropy |
| logarithm_glcm_SumSquares | logarithm_glcm_SumSquares |
| **logarithm_gldm_DependenceEntropy** | logarithm_gldm_DependenceEntropy |
| logarithm_gldm_DependenceNonUniformity | logarithm_gldm_DependenceNonUniformity |
| logarithm_gldm_DependenceNonUniformityNormalized | logarithm_gldm_DependenceNonUniformityNormalized |
| logarithm_gldm_DependenceVariance | logarithm_gldm_DependenceVariance |
| logarithm_gldm_GrayLevelNonUniformity | logarithm_gldm_GrayLevelNonUniformity |
| logarithm_gldm_GrayLevelVariance | logarithm_gldm_GrayLevelVariance |
| **logarithm_gldm_HighGrayLevelEmphasis** | logarithm_gldm_HighGrayLevelEmphasis |
| **logarithm_gldm_LargeDependenceEmphasis** | logarithm_gldm_LargeDependenceEmphasis |
| logarithm_gldm_LargeDependenceHighGrayLevelEmphasis | logarithm_gldm_LargeDependenceHighGrayLevelEmphasis |
| **logarithm_gldm_LargeDependenceLowGrayLevelEmphasis** | logarithm_gldm_LargeDependenceLowGrayLevelEmphasis |
| **logarithm_gldm_LowGrayLevelEmphasis** | logarithm_gldm_LowGrayLevelEmphasis |
| **logarithm_gldm_SmallDependenceEmphasis** | logarithm_gldm_SmallDependenceEmphasis |
| logarithm_gldm_SmallDependenceHighGrayLevelEmphasis | logarithm_gldm_SmallDependenceHighGrayLevelEmphasis |
| **logarithm_gldm_SmallDependenceLowGrayLevelEmphasis** | logarithm_gldm_SmallDependenceLowGrayLevelEmphasis |
| logarithm_glrlm_GrayLevelNonUniformity | logarithm_glrlm_GrayLevelNonUniformity |
| **logarithm_glrlm_GrayLevelNonUniformityNormalized** | **logarithm_glrlm_GrayLevelNonUniformityNormalized** |
| **logarithm_glrlm_GrayLevelVariance** | logarithm_glrlm_GrayLevelVariance |
| **logarithm_glrlm_HighGrayLevelRunEmphasis** | logarithm_glrlm_HighGrayLevelRunEmphasis |
| logarithm_glrlm_LongRunEmphasis | logarithm_glrlm_LongRunEmphasis |
| logarithm_glrlm_LongRunHighGrayLevelEmphasis | logarithm_glrlm_LongRunHighGrayLevelEmphasis |
| **logarithm_glrlm_LongRunLowGrayLevelEmphasis** | logarithm_glrlm_LongRunLowGrayLevelEmphasis |
| **logarithm_glrlm_LowGrayLevelRunEmphasis** | logarithm_glrlm_LowGrayLevelRunEmphasis |
| **logarithm_glrlm_RunEntropy** | logarithm_glrlm_RunEntropy |
| logarithm_glrlm_RunLengthNonUniformity | logarithm_glrlm_RunLengthNonUniformity |
| **logarithm_glrlm_RunLengthNonUniformityNormalized** | logarithm_glrlm_RunLengthNonUniformityNormalized |
| **logarithm_glrlm_RunPercentage** | logarithm_glrlm_RunPercentage |
| logarithm_glrlm_RunVariance | logarithm_glrlm_RunVariance |
| **logarithm_glrlm_ShortRunEmphasis** | **logarithm_glrlm_ShortRunEmphasis** |
| **logarithm_glrlm_ShortRunHighGrayLevelEmphasis** | logarithm_glrlm_ShortRunHighGrayLevelEmphasis |
| **logarithm_glrlm_ShortRunLowGrayLevelEmphasis** | logarithm_glrlm_ShortRunLowGrayLevelEmphasis |
| logarithm_glszm_GrayLevelNonUniformity | logarithm_glszm_GrayLevelNonUniformity |
| **logarithm_glszm_GrayLevelNonUniformityNormalized** | logarithm_glszm_GrayLevelNonUniformityNormalized |
| **logarithm_glszm_GrayLevelVariance** | logarithm_glszm_GrayLevelVariance |
| **logarithm_glszm_HighGrayLevelZoneEmphasis** | logarithm_glszm_HighGrayLevelZoneEmphasis |
| logarithm_glszm_LargeAreaEmphasis | logarithm_glszm_LargeAreaEmphasis |
| logarithm_glszm_LargeAreaHighGrayLevelEmphasis | logarithm_glszm_LargeAreaHighGrayLevelEmphasis |
| logarithm_glszm_LargeAreaLowGrayLevelEmphasis | logarithm_glszm_LargeAreaLowGrayLevelEmphasis |
| **logarithm_glszm_LowGrayLevelZoneEmphasis** | logarithm_glszm_LowGrayLevelZoneEmphasis |
| logarithm_glszm_SizeZoneNonUniformity | logarithm_glszm_SizeZoneNonUniformity |
| **logarithm_glszm_SizeZoneNonUniformityNormalized** | logarithm_glszm_SizeZoneNonUniformityNormalized |
| **logarithm_glszm_SmallAreaEmphasis** | **logarithm_glszm_SmallAreaEmphasis** |
| **logarithm_glszm_SmallAreaHighGrayLevelEmphasis** | logarithm_glszm_SmallAreaHighGrayLevelEmphasis |
| **logarithm_glszm_SmallAreaLowGrayLevelEmphasis** | logarithm_glszm_SmallAreaLowGrayLevelEmphasis |
| **logarithm_glszm_ZoneEntropy** | **logarithm_glszm_ZoneEntropy** |
| **logarithm_glszm_ZonePercentage** | logarithm_glszm_ZonePercentage |
| logarithm_glszm_ZoneVariance | logarithm_glszm_ZoneVariance |
| logarithm_ngtdm_Busyness | logarithm_ngtdm_Busyness |
| **logarithm_ngtdm_Coarseness** | **logarithm_ngtdm_Coarseness** |
| logarithm_ngtdm_Complexity | logarithm_ngtdm_Complexity |
| **logarithm_ngtdm_Contrast** | logarithm_ngtdm_Contrast |
| logarithm_ngtdm_Strength | logarithm_ngtdm_Strength |
| original_glcm_Autocorrelation | original_glcm_Autocorrelation |
| original_glcm_ClusterProminence | original_glcm_ClusterProminence |
| original_glcm_ClusterShade | original_glcm_ClusterShade |
| original_glcm_ClusterTendency | original_glcm_ClusterTendency |
| original_glcm_Contrast | original_glcm_Contrast |
| original_glcm_Correlation | original_glcm_Correlation |
| original_glcm_DifferenceAverage | original_glcm_DifferenceAverage |
| original_glcm_DifferenceEntropy | original_glcm_DifferenceEntropy |
| original_glcm_DifferenceVariance | original_glcm_DifferenceVariance |
| original_glcm_Id | original_glcm_Id |
| original_glcm_Idm | original_glcm_Idm |
| original_glcm_Idmn | original_glcm_Idmn |
| original_glcm_Idn | original_glcm_Idn |
| original_glcm_Imc1 | original_glcm_Imc1 |
| original_glcm_Imc2 | original_glcm_Imc2 |
| original_glcm_InverseVariance | original_glcm_InverseVariance |
| original_glcm_JointAverage | original_glcm_JointAverage |
| original_glcm_JointEnergy | original_glcm_JointEnergy |
| original_glcm_JointEntropy | original_glcm_JointEntropy |
| original_glcm_MaximumProbability | original_glcm_MaximumProbability |
| original_glcm_SumAverage | original_glcm_SumAverage |
| original_glcm_SumEntropy | original_glcm_SumEntropy |
| original_glcm_SumSquares | original_glcm_SumSquares |
| original_gldm_DependenceEntropy | original_gldm_DependenceEntropy |
| original_gldm_DependenceNonUniformity | original_gldm_DependenceNonUniformity |
| original_gldm_DependenceNonUniformityNormalized | original_gldm_DependenceNonUniformityNormalized |
| original_gldm_DependenceVariance | original_gldm_DependenceVariance |
| original_gldm_GrayLevelNonUniformity | original_gldm_GrayLevelNonUniformity |
| original_gldm_GrayLevelVariance | original_gldm_GrayLevelVariance |
| original_gldm_HighGrayLevelEmphasis | original_gldm_HighGrayLevelEmphasis |
| original_gldm_LargeDependenceEmphasis | original_gldm_LargeDependenceEmphasis |
| original_gldm_LargeDependenceHighGrayLevelEmphasis | original_gldm_LargeDependenceHighGrayLevelEmphasis |
| original_gldm_LargeDependenceLowGrayLevelEmphasis | original_gldm_LargeDependenceLowGrayLevelEmphasis |
| original_gldm_LowGrayLevelEmphasis | original_gldm_LowGrayLevelEmphasis |
| original_gldm_SmallDependenceEmphasis | original_gldm_SmallDependenceEmphasis |
| original_gldm_SmallDependenceHighGrayLevelEmphasis | original_gldm_SmallDependenceHighGrayLevelEmphasis |
| **original_gldm_SmallDependenceLowGrayLevelEmphasis** | original_gldm_SmallDependenceLowGrayLevelEmphasis |
| original_glrlm_GrayLevelNonUniformity | original_glrlm_GrayLevelNonUniformity |
| original_glrlm_GrayLevelNonUniformityNormalized | original_glrlm_GrayLevelNonUniformityNormalized |
| original_glrlm_GrayLevelVariance | original_glrlm_GrayLevelVariance |
| original_glrlm_HighGrayLevelRunEmphasis | original_glrlm_HighGrayLevelRunEmphasis |
| original_glrlm_LongRunEmphasis | original_glrlm_LongRunEmphasis |
| original_glrlm_LongRunHighGrayLevelEmphasis | original_glrlm_LongRunHighGrayLevelEmphasis |
| original_glrlm_LongRunLowGrayLevelEmphasis | original_glrlm_LongRunLowGrayLevelEmphasis |
| original_glrlm_LowGrayLevelRunEmphasis | original_glrlm_LowGrayLevelRunEmphasis |
| original_glrlm_RunEntropy | original_glrlm_RunEntropy |
| original_glrlm_RunLengthNonUniformity | original_glrlm_RunLengthNonUniformity |
| original_glrlm_RunLengthNonUniformityNormalized | original_glrlm_RunLengthNonUniformityNormalized |
| original_glrlm_RunPercentage | original_glrlm_RunPercentage |
| original_glrlm_RunVariance | original_glrlm_RunVariance |
| original_glrlm_ShortRunEmphasis | original_glrlm_ShortRunEmphasis |
| original_glrlm_ShortRunHighGrayLevelEmphasis | original_glrlm_ShortRunHighGrayLevelEmphasis |
| original_glrlm_ShortRunLowGrayLevelEmphasis | original_glrlm_ShortRunLowGrayLevelEmphasis |
| original_glszm_GrayLevelNonUniformity | original_glszm_GrayLevelNonUniformity |
| original_glszm_GrayLevelNonUniformityNormalized | original_glszm_GrayLevelNonUniformityNormalized |
| original_glszm_GrayLevelVariance | original_glszm_GrayLevelVariance |
| original_glszm_HighGrayLevelZoneEmphasis | original_glszm_HighGrayLevelZoneEmphasis |
| original_glszm_LargeAreaEmphasis | original_glszm_LargeAreaEmphasis |
| original_glszm_LargeAreaHighGrayLevelEmphasis | original_glszm_LargeAreaHighGrayLevelEmphasis |
| original_glszm_LargeAreaLowGrayLevelEmphasis | original_glszm_LargeAreaLowGrayLevelEmphasis |
| original_glszm_LowGrayLevelZoneEmphasis | original_glszm_LowGrayLevelZoneEmphasis |
| original_glszm_SizeZoneNonUniformity | original_glszm_SizeZoneNonUniformity |
| original_glszm_SizeZoneNonUniformityNormalized | original_glszm_SizeZoneNonUniformityNormalized |
| **original_glszm_SmallAreaEmphasis** | **original_glszm_SmallAreaEmphasis** |
| **original_glszm_SmallAreaHighGrayLevelEmphasis** | original_glszm_SmallAreaHighGrayLevelEmphasis |
| original_glszm_SmallAreaLowGrayLevelEmphasis | original_glszm_SmallAreaLowGrayLevelEmphasis |
| **original_glszm_ZoneEntropy** | original_glszm_ZoneEntropy |
| **original_glszm_ZonePercentage** | original_glszm_ZonePercentage |
| original_glszm_ZoneVariance | original_glszm_ZoneVariance |
| original_ngtdm_Busyness | original_ngtdm_Busyness |
| **original_ngtdm_Coarseness** | original_ngtdm_Coarseness |
| **original_ngtdm_Complexity** | original_ngtdm_Complexity |
| **original_ngtdm_Contrast** | original_ngtdm_Contrast |
| **original_ngtdm_Strength** | original_ngtdm_Strength |
| square_glcm_Autocorrelation | square_glcm_Autocorrelation |
| square_glcm_ClusterProminence | square_glcm_ClusterProminence |
| square_glcm_ClusterShade | square_glcm_ClusterShade |
| square_glcm_ClusterTendency | square_glcm_ClusterTendency |
| square_glcm_Contrast | square_glcm_Contrast |
| square_glcm_Correlation | square_glcm_Correlation |
| square_glcm_DifferenceAverage | square_glcm_DifferenceAverage |
| square_glcm_DifferenceEntropy | square_glcm_DifferenceEntropy |
| square_glcm_DifferenceVariance | square_glcm_DifferenceVariance |
| square_glcm_Id | square_glcm_Id |
| square_glcm_Idm | square_glcm_Idm |
| **square_glcm_Idmn** | square_glcm_Idmn |
| square_glcm_Idn | square_glcm_Idn |
| square_glcm_Imc1 | square_glcm_Imc1 |
| square_glcm_Imc2 | square_glcm_Imc2 |
| square_glcm_InverseVariance | square_glcm_InverseVariance |
| square_glcm_JointAverage | square_glcm_JointAverage |
| square_glcm_JointEnergy | square_glcm_JointEnergy |
| square_glcm_JointEntropy | square_glcm_JointEntropy |
| square_glcm_MaximumProbability | square_glcm_MaximumProbability |
| square_glcm_SumAverage | square_glcm_SumAverage |
| square_glcm_SumEntropy | square_glcm_SumEntropy |
| square_glcm_SumSquares | square_glcm_SumSquares |
| **square_gldm_DependenceEntropy** | square_gldm_DependenceEntropy |
| square_gldm_DependenceNonUniformity | square_gldm_DependenceNonUniformity |
| **square_gldm_DependenceNonUniformityNormalized** | square_gldm_DependenceNonUniformityNormalized |
| **square_gldm_DependenceVariance** | square_gldm_DependenceVariance |
| square_gldm_GrayLevelNonUniformity | square_gldm_GrayLevelNonUniformity |
| square_gldm_GrayLevelVariance | square_gldm_GrayLevelVariance |
| square_gldm_HighGrayLevelEmphasis | square_gldm_HighGrayLevelEmphasis |
| **square_gldm_LargeDependenceEmphasis** | square_gldm_LargeDependenceEmphasis |
| **square_gldm_LargeDependenceHighGrayLevelEmphasis** | square_gldm_LargeDependenceHighGrayLevelEmphasis |
| **square_gldm_LargeDependenceLowGrayLevelEmphasis** | square_gldm_LargeDependenceLowGrayLevelEmphasis |
| square_gldm_LowGrayLevelEmphasis | square_gldm_LowGrayLevelEmphasis |
| **square_gldm_SmallDependenceEmphasis** | square_gldm_SmallDependenceEmphasis |
| **square_gldm_SmallDependenceHighGrayLevelEmphasis** | square_gldm_SmallDependenceHighGrayLevelEmphasis |
| **square_gldm_SmallDependenceLowGrayLevelEmphasis** | square_gldm_SmallDependenceLowGrayLevelEmphasis |
| square_glrlm_GrayLevelNonUniformity | square_glrlm_GrayLevelNonUniformity |
| square_glrlm_GrayLevelNonUniformityNormalized | square_glrlm_GrayLevelNonUniformityNormalized |
| square_glrlm_GrayLevelVariance | square_glrlm_GrayLevelVariance |
| square_glrlm_HighGrayLevelRunEmphasis | square_glrlm_HighGrayLevelRunEmphasis |
| square_glrlm_LongRunEmphasis | square_glrlm_LongRunEmphasis |
| square_glrlm_LongRunHighGrayLevelEmphasis | square_glrlm_LongRunHighGrayLevelEmphasis |
| square_glrlm_LongRunLowGrayLevelEmphasis | square_glrlm_LongRunLowGrayLevelEmphasis |
| square_glrlm_LowGrayLevelRunEmphasis | square_glrlm_LowGrayLevelRunEmphasis |
| **square_glrlm_RunEntropy** | square_glrlm_RunEntropy |
| square_glrlm_RunLengthNonUniformity | square_glrlm_RunLengthNonUniformity |
| **square_glrlm_RunLengthNonUniformityNormalized** | **square_glrlm_RunLengthNonUniformityNormalized** |
| **square_glrlm_RunPercentage** | square_glrlm_RunPercentage |
| square_glrlm_RunVariance | square_glrlm_RunVariance |
| **square_glrlm_ShortRunEmphasis** | square_glrlm_ShortRunEmphasis |
| square_glrlm_ShortRunHighGrayLevelEmphasis | square_glrlm_ShortRunHighGrayLevelEmphasis |
| **square_glrlm_ShortRunLowGrayLevelEmphasis** | square_glrlm_ShortRunLowGrayLevelEmphasis |
| square_glszm_GrayLevelNonUniformity | square_glszm_GrayLevelNonUniformity |
| square_glszm_GrayLevelNonUniformityNormalized | square_glszm_GrayLevelNonUniformityNormalized |
| square_glszm_GrayLevelVariance | square_glszm_GrayLevelVariance |
| square_glszm_HighGrayLevelZoneEmphasis | square_glszm_HighGrayLevelZoneEmphasis |
| square_glszm_LargeAreaEmphasis | square_glszm_LargeAreaEmphasis |
| square_glszm_LargeAreaHighGrayLevelEmphasis | square_glszm_LargeAreaHighGrayLevelEmphasis |
| square_glszm_LargeAreaLowGrayLevelEmphasis | square_glszm_LargeAreaLowGrayLevelEmphasis |
| square_glszm_LowGrayLevelZoneEmphasis | square_glszm_LowGrayLevelZoneEmphasis |
| square_glszm_SizeZoneNonUniformity | square_glszm_SizeZoneNonUniformity |
| square_glszm_SizeZoneNonUniformityNormalized | square_glszm_SizeZoneNonUniformityNormalized |
| square_glszm_SmallAreaEmphasis | square_glszm_SmallAreaEmphasis |
| square_glszm_SmallAreaHighGrayLevelEmphasis | square_glszm_SmallAreaHighGrayLevelEmphasis |
| square_glszm_SmallAreaLowGrayLevelEmphasis | square_glszm_SmallAreaLowGrayLevelEmphasis |
| square_glszm_ZoneEntropy | square_glszm_ZoneEntropy |
| **square_glszm_ZonePercentage** | square_glszm_ZonePercentage |
| square_glszm_ZoneVariance | square_glszm_ZoneVariance |
| square_ngtdm_Busyness | square_ngtdm_Busyness |
| square_ngtdm_Coarseness | square_ngtdm_Coarseness |
| square_ngtdm_Complexity | square_ngtdm_Complexity |
| square_ngtdm_Strength | square_ngtdm_Strength |
| **squareroot_glcm_Autocorrelation** | squareroot_glcm_Autocorrelation |
| squareroot_glcm_ClusterProminence | squareroot_glcm_ClusterProminence |
| squareroot_glcm_ClusterShade | squareroot_glcm_ClusterShade |
| **squareroot_glcm_ClusterTendency** | squareroot_glcm_ClusterTendency |
| **squareroot_glcm_Contrast** | squareroot_glcm_Contrast |
| squareroot_glcm_Correlation | squareroot_glcm_Correlation |
| **squareroot_glcm_DifferenceAverage** | squareroot_glcm_DifferenceAverage |
| **squareroot_glcm_DifferenceEntropy** | squareroot_glcm_DifferenceEntropy |
| **squareroot_glcm_DifferenceVariance** | squareroot_glcm_DifferenceVariance |
| **squareroot_glcm_Id** | squareroot_glcm_Id |
| **squareroot_glcm_Idm** | squareroot_glcm_Idm |
| squareroot_glcm_Idmn | squareroot_glcm_Idmn |
| squareroot_glcm_Idn | squareroot_glcm_Idn |
| squareroot_glcm_Imc1 | squareroot_glcm_Imc1 |
| **squareroot_glcm_Imc2** | squareroot_glcm_Imc2 |
| **squareroot_glcm_InverseVariance** | squareroot_glcm_InverseVariance |
| **squareroot_glcm_JointAverage** | squareroot_glcm_JointAverage |
| **squareroot_glcm_JointEnergy** | squareroot_glcm_JointEnergy |
| **squareroot_glcm_JointEntropy** | squareroot_glcm_JointEntropy |
| **squareroot_glcm_MaximumProbability** | squareroot_glcm_MaximumProbability |
| **squareroot_glcm_SumAverage** | squareroot_glcm_SumAverage |
| **squareroot_glcm_SumEntropy** | squareroot_glcm_SumEntropy |
| **squareroot_glcm_SumSquares** | squareroot_glcm_SumSquares |
| **squareroot_gldm_DependenceEntropy** | squareroot_gldm_DependenceEntropy |
| squareroot_gldm_DependenceNonUniformity | squareroot_gldm_DependenceNonUniformity |
| squareroot_gldm_DependenceNonUniformityNormalized | squareroot_gldm_DependenceNonUniformityNormalized |
| **squareroot_gldm_DependenceVariance** | squareroot_gldm_DependenceVariance |
| squareroot_gldm_GrayLevelNonUniformity | squareroot_gldm_GrayLevelNonUniformity |
| **squareroot_gldm_GrayLevelVariance** | squareroot_gldm_GrayLevelVariance |
| **squareroot_gldm_HighGrayLevelEmphasis** | squareroot_gldm_HighGrayLevelEmphasis |
| **squareroot_gldm_LargeDependenceEmphasis** | squareroot_gldm_LargeDependenceEmphasis |
| squareroot_gldm_LargeDependenceHighGrayLevelEmphasis | squareroot_gldm_LargeDependenceHighGrayLevelEmphasis |
| **squareroot_gldm_LargeDependenceLowGrayLevelEmphasis** | squareroot_gldm_LargeDependenceLowGrayLevelEmphasis |
| **squareroot_gldm_LowGrayLevelEmphasis** | squareroot_gldm_LowGrayLevelEmphasis |
| **squareroot_gldm_SmallDependenceEmphasis** | squareroot_gldm_SmallDependenceEmphasis |
| squareroot_gldm_SmallDependenceHighGrayLevelEmphasis | squareroot_gldm_SmallDependenceHighGrayLevelEmphasis |
| **squareroot_gldm_SmallDependenceLowGrayLevelEmphasis** | squareroot_gldm_SmallDependenceLowGrayLevelEmphasis |
| squareroot_glrlm_GrayLevelNonUniformity | squareroot_glrlm_GrayLevelNonUniformity |
| **squareroot_glrlm_GrayLevelNonUniformityNormalized** | **squareroot_glrlm_GrayLevelNonUniformityNormalized** |
| **squareroot_glrlm_GrayLevelVariance** | squareroot_glrlm_GrayLevelVariance |
| **squareroot_glrlm_HighGrayLevelRunEmphasis** | squareroot_glrlm_HighGrayLevelRunEmphasis |
| squareroot_glrlm_LongRunEmphasis | squareroot_glrlm_LongRunEmphasis |
| squareroot_glrlm_LongRunHighGrayLevelEmphasis | squareroot_glrlm_LongRunHighGrayLevelEmphasis |
| **squareroot_glrlm_LongRunLowGrayLevelEmphasis** | squareroot_glrlm_LongRunLowGrayLevelEmphasis |
| **squareroot_glrlm_LowGrayLevelRunEmphasis** | squareroot_glrlm_LowGrayLevelRunEmphasis |
| **squareroot_glrlm_RunEntropy** | squareroot_glrlm_RunEntropy |
| squareroot_glrlm_RunLengthNonUniformity | squareroot_glrlm_RunLengthNonUniformity |
| **squareroot_glrlm_RunLengthNonUniformityNormalized** | squareroot_glrlm_RunLengthNonUniformityNormalized |
| **squareroot_glrlm_RunPercentage** | squareroot_glrlm_RunPercentage |
| squareroot_glrlm_RunVariance | squareroot_glrlm_RunVariance |
| **squareroot_glrlm_ShortRunEmphasis** | squareroot_glrlm_ShortRunEmphasis |
| **squareroot_glrlm_ShortRunHighGrayLevelEmphasis** | squareroot_glrlm_ShortRunHighGrayLevelEmphasis |
| **squareroot_glrlm_ShortRunLowGrayLevelEmphasis** | squareroot_glrlm_ShortRunLowGrayLevelEmphasis |
| squareroot_glszm_GrayLevelNonUniformity | squareroot_glszm_GrayLevelNonUniformity |
| **squareroot_glszm_GrayLevelNonUniformityNormalized** | squareroot_glszm_GrayLevelNonUniformityNormalized |
| **squareroot_glszm_GrayLevelVariance** | squareroot_glszm_GrayLevelVariance |
| **squareroot_glszm_HighGrayLevelZoneEmphasis** | squareroot_glszm_HighGrayLevelZoneEmphasis |
| squareroot_glszm_LargeAreaEmphasis | squareroot_glszm_LargeAreaEmphasis |
| squareroot_glszm_LargeAreaHighGrayLevelEmphasis | squareroot_glszm_LargeAreaHighGrayLevelEmphasis |
| squareroot_glszm_LargeAreaLowGrayLevelEmphasis | squareroot_glszm_LargeAreaLowGrayLevelEmphasis |
| **squareroot_glszm_LowGrayLevelZoneEmphasis** | squareroot_glszm_LowGrayLevelZoneEmphasis |
| squareroot_glszm_SizeZoneNonUniformity | squareroot_glszm_SizeZoneNonUniformity |
| **squareroot_glszm_SizeZoneNonUniformityNormalized** | **squareroot_glszm_SizeZoneNonUniformityNormalized** |
| **squareroot_glszm_SmallAreaEmphasis** | **squareroot_glszm_SmallAreaEmphasis** |
| **squareroot_glszm_SmallAreaHighGrayLevelEmphasis** | squareroot_glszm_SmallAreaHighGrayLevelEmphasis |
| **squareroot_glszm_SmallAreaLowGrayLevelEmphasis** | squareroot_glszm_SmallAreaLowGrayLevelEmphasis |
| **squareroot_glszm_ZoneEntropy** | squareroot_glszm_ZoneEntropy |
| **squareroot_glszm_ZonePercentage** | squareroot_glszm_ZonePercentage |
| squareroot_glszm_ZoneVariance | squareroot_glszm_ZoneVariance |
| squareroot_ngtdm_Busyness | squareroot_ngtdm_Busyness |
| **squareroot_ngtdm_Coarseness** | **squareroot_ngtdm_Coarseness** |
| squareroot_ngtdm_Complexity | squareroot_ngtdm_Complexity |
| **squareroot_ngtdm_Contrast** | squareroot_ngtdm_Contrast |
| squareroot_ngtdm_Strength | squareroot_ngtdm_Strength |
| wavelet_HHH_glcm_Autocorrelation | wavelet_HHH_glcm_Autocorrelation |
| wavelet_HHH_glcm_ClusterProminence | wavelet_HHH_glcm_ClusterProminence |
| wavelet_HHH_glcm_ClusterShade | wavelet_HHH_glcm_ClusterShade |
| wavelet_HHH_glcm_ClusterTendency | wavelet_HHH_glcm_ClusterTendency |
| wavelet_HHH_glcm_Contrast | wavelet_HHH_glcm_Contrast |
| wavelet_HHH_glcm_Correlation | wavelet_HHH_glcm_Correlation |
| wavelet_HHH_glcm_DifferenceAverage | wavelet_HHH_glcm_DifferenceAverage |
| wavelet_HHH_glcm_DifferenceEntropy | wavelet_HHH_glcm_DifferenceEntropy |
| wavelet_HHH_glcm_DifferenceVariance | wavelet_HHH_glcm_DifferenceVariance |
| wavelet_HHH_glcm_Id | wavelet_HHH_glcm_Id |
| wavelet_HHH_glcm_Idm | wavelet_HHH_glcm_Idm |
| **wavelet_HHH_glcm_Idmn** | wavelet_HHH_glcm_Idmn |
| wavelet_HHH_glcm_Idn | wavelet_HHH_glcm_Idn |
| wavelet_HHH_glcm_Imc1 | wavelet_HHH_glcm_Imc1 |
| wavelet_HHH_glcm_Imc2 | wavelet_HHH_glcm_Imc2 |
| wavelet_HHH_glcm_InverseVariance | wavelet_HHH_glcm_InverseVariance |
| wavelet_HHH_glcm_JointAverage | wavelet_HHH_glcm_JointAverage |
| wavelet_HHH_glcm_JointEnergy | wavelet_HHH_glcm_JointEnergy |
| wavelet_HHH_glcm_JointEntropy | wavelet_HHH_glcm_JointEntropy |
| wavelet_HHH_glcm_MaximumProbability | wavelet_HHH_glcm_MaximumProbability |
| wavelet_HHH_glcm_SumAverage | wavelet_HHH_glcm_SumAverage |
| wavelet_HHH_glcm_SumEntropy | wavelet_HHH_glcm_SumEntropy |
| wavelet_HHH_glcm_SumSquares | wavelet_HHH_glcm_SumSquares |
| wavelet_HHH_gldm_DependenceEntropy | wavelet_HHH_gldm_DependenceEntropy |
| wavelet_HHH_gldm_DependenceNonUniformity | wavelet_HHH_gldm_DependenceNonUniformity |
| wavelet_HHH_gldm_DependenceNonUniformityNormalized | wavelet_HHH_gldm_DependenceNonUniformityNormalized |
| wavelet_HHH_gldm_DependenceVariance | wavelet_HHH_gldm_DependenceVariance |
| wavelet_HHH_gldm_GrayLevelNonUniformity | wavelet_HHH_gldm_GrayLevelNonUniformity |
| wavelet_HHH_gldm_GrayLevelVariance | wavelet_HHH_gldm_GrayLevelVariance |
| wavelet_HHH_gldm_HighGrayLevelEmphasis | wavelet_HHH_gldm_HighGrayLevelEmphasis |
| **wavelet_HHH_gldm_LargeDependenceEmphasis** | wavelet_HHH_gldm_LargeDependenceEmphasis |
| wavelet_HHH_gldm_LargeDependenceHighGrayLevelEmphasis | wavelet_HHH_gldm_LargeDependenceHighGrayLevelEmphasis |
| wavelet_HHH_gldm_LargeDependenceLowGrayLevelEmphasis | wavelet_HHH_gldm_LargeDependenceLowGrayLevelEmphasis |
| **wavelet_HHH_gldm_LowGrayLevelEmphasis** | wavelet_HHH_gldm_LowGrayLevelEmphasis |
| **wavelet_HHH_gldm_SmallDependenceEmphasis** | wavelet_HHH_gldm_SmallDependenceEmphasis |
| wavelet_HHH_gldm_SmallDependenceHighGrayLevelEmphasis | wavelet_HHH_gldm_SmallDependenceHighGrayLevelEmphasis |
| **wavelet_HHH_gldm_SmallDependenceLowGrayLevelEmphasis** | **wavelet_HHH_gldm_SmallDependenceLowGrayLevelEmphasis** |
| wavelet_HHH_glrlm_GrayLevelNonUniformity | wavelet_HHH_glrlm_GrayLevelNonUniformity |
| wavelet_HHH_glrlm_GrayLevelNonUniformityNormalized | wavelet_HHH_glrlm_GrayLevelNonUniformityNormalized |
| wavelet_HHH_glrlm_GrayLevelVariance | wavelet_HHH_glrlm_GrayLevelVariance |
| wavelet_HHH_glrlm_HighGrayLevelRunEmphasis | wavelet_HHH_glrlm_HighGrayLevelRunEmphasis |
| **wavelet_HHH_glrlm_LongRunEmphasis** | wavelet_HHH_glrlm_LongRunEmphasis |
| wavelet_HHH_glrlm_LongRunHighGrayLevelEmphasis | wavelet_HHH_glrlm_LongRunHighGrayLevelEmphasis |
| wavelet_HHH_glrlm_LongRunLowGrayLevelEmphasis | wavelet_HHH_glrlm_LongRunLowGrayLevelEmphasis |
| **wavelet_HHH_glrlm_LowGrayLevelRunEmphasis** | wavelet_HHH_glrlm_LowGrayLevelRunEmphasis |
| **wavelet_HHH_glrlm_RunEntropy** | wavelet_HHH_glrlm_RunEntropy |
| wavelet_HHH_glrlm_RunLengthNonUniformity | wavelet_HHH_glrlm_RunLengthNonUniformity |
| **wavelet_HHH_glrlm_RunLengthNonUniformityNormalized** | wavelet_HHH_glrlm_RunLengthNonUniformityNormalized |
| **wavelet_HHH_glrlm_RunPercentage** | wavelet_HHH_glrlm_RunPercentage |
| **wavelet_HHH_glrlm_RunVariance** | wavelet_HHH_glrlm_RunVariance |
| **wavelet_HHH_glrlm_ShortRunEmphasis** | wavelet_HHH_glrlm_ShortRunEmphasis |
| wavelet_HHH_glrlm_ShortRunHighGrayLevelEmphasis | wavelet_HHH_glrlm_ShortRunHighGrayLevelEmphasis |
| **wavelet_HHH_glrlm_ShortRunLowGrayLevelEmphasis** | wavelet_HHH_glrlm_ShortRunLowGrayLevelEmphasis |
| wavelet_HHH_glszm_GrayLevelNonUniformity | wavelet_HHH_glszm_GrayLevelNonUniformity |
| wavelet_HHH_glszm_GrayLevelNonUniformityNormalized | wavelet_HHH_glszm_GrayLevelNonUniformityNormalized |
| wavelet_HHH_glszm_GrayLevelVariance | wavelet_HHH_glszm_GrayLevelVariance |
| wavelet_HHH_glszm_HighGrayLevelZoneEmphasis | wavelet_HHH_glszm_HighGrayLevelZoneEmphasis |
| wavelet_HHH_glszm_LargeAreaEmphasis | wavelet_HHH_glszm_LargeAreaEmphasis |
| wavelet_HHH_glszm_LargeAreaHighGrayLevelEmphasis | wavelet_HHH_glszm_LargeAreaHighGrayLevelEmphasis |
| wavelet_HHH_glszm_LargeAreaLowGrayLevelEmphasis | wavelet_HHH_glszm_LargeAreaLowGrayLevelEmphasis |
| wavelet_HHH_glszm_LowGrayLevelZoneEmphasis | wavelet_HHH_glszm_LowGrayLevelZoneEmphasis |
| wavelet_HHH_glszm_SizeZoneNonUniformity | wavelet_HHH_glszm_SizeZoneNonUniformity |
| wavelet_HHH_glszm_SizeZoneNonUniformityNormalized | wavelet_HHH_glszm_SizeZoneNonUniformityNormalized |
| **wavelet_HHH_glszm_SmallAreaEmphasis** | **wavelet_HHH_glszm_SmallAreaEmphasis** |
| wavelet_HHH_glszm_SmallAreaHighGrayLevelEmphasis | wavelet_HHH_glszm_SmallAreaHighGrayLevelEmphasis |
| wavelet_HHH_glszm_SmallAreaLowGrayLevelEmphasis | wavelet_HHH_glszm_SmallAreaLowGrayLevelEmphasis |
| **wavelet_HHH_glszm_ZoneEntropy** | wavelet_HHH_glszm_ZoneEntropy |
| **wavelet_HHH_glszm_ZonePercentage** | wavelet_HHH_glszm_ZonePercentage |
| wavelet_HHH_glszm_ZoneVariance | wavelet_HHH_glszm_ZoneVariance |
| wavelet_HHH_ngtdm_Busyness | wavelet_HHH_ngtdm_Busyness |
| **wavelet_HHH_ngtdm_Coarseness** | **wavelet_HHH_ngtdm_Coarseness** |
| wavelet_HHH_ngtdm_Complexity | wavelet_HHH_ngtdm_Complexity |
| **wavelet_HHH_ngtdm_Contrast** | **wavelet_HHH_ngtdm_Contrast** |
| wavelet_HHH_ngtdm_Strength | wavelet_HHH_ngtdm_Strength |
| wavelet_HHL_glcm_Autocorrelation | wavelet_HHL_glcm_Autocorrelation |
| wavelet_HHL_glcm_ClusterProminence | wavelet_HHL_glcm_ClusterProminence |
| wavelet_HHL_glcm_ClusterShade | wavelet_HHL_glcm_ClusterShade |
| wavelet_HHL_glcm_ClusterTendency | wavelet_HHL_glcm_ClusterTendency |
| wavelet_HHL_glcm_Contrast | wavelet_HHL_glcm_Contrast |
| wavelet_HHL_glcm_Correlation | wavelet_HHL_glcm_Correlation |
| wavelet_HHL_glcm_DifferenceAverage | wavelet_HHL_glcm_DifferenceAverage |
| wavelet_HHL_glcm_DifferenceEntropy | wavelet_HHL_glcm_DifferenceEntropy |
| wavelet_HHL_glcm_DifferenceVariance | wavelet_HHL_glcm_DifferenceVariance |
| wavelet_HHL_glcm_Id | wavelet_HHL_glcm_Id |
| wavelet_HHL_glcm_Idm | wavelet_HHL_glcm_Idm |
| wavelet_HHL_glcm_Idmn | wavelet_HHL_glcm_Idmn |
| **wavelet_HHL_glcm_Idn** | wavelet_HHL_glcm_Idn |
| wavelet_HHL_glcm_Imc1 | wavelet_HHL_glcm_Imc1 |
| wavelet_HHL_glcm_Imc2 | wavelet_HHL_glcm_Imc2 |
| wavelet_HHL_glcm_InverseVariance | wavelet_HHL_glcm_InverseVariance |
| wavelet_HHL_glcm_JointAverage | wavelet_HHL_glcm_JointAverage |
| wavelet_HHL_glcm_JointEnergy | wavelet_HHL_glcm_JointEnergy |
| wavelet_HHL_glcm_JointEntropy | wavelet_HHL_glcm_JointEntropy |
| wavelet_HHL_glcm_MaximumProbability | wavelet_HHL_glcm_MaximumProbability |
| wavelet_HHL_glcm_SumAverage | wavelet_HHL_glcm_SumAverage |
| wavelet_HHL_glcm_SumEntropy | wavelet_HHL_glcm_SumEntropy |
| wavelet_HHL_glcm_SumSquares | wavelet_HHL_glcm_SumSquares |
| wavelet_HHL_gldm_DependenceEntropy | wavelet_HHL_gldm_DependenceEntropy |
| wavelet_HHL_gldm_DependenceNonUniformity | wavelet_HHL_gldm_DependenceNonUniformity |
| wavelet_HHL_gldm_DependenceNonUniformityNormalized | wavelet_HHL_gldm_DependenceNonUniformityNormalized |
| wavelet_HHL_gldm_DependenceVariance | wavelet_HHL_gldm_DependenceVariance |
| wavelet_HHL_gldm_GrayLevelNonUniformity | wavelet_HHL_gldm_GrayLevelNonUniformity |
| wavelet_HHL_gldm_GrayLevelVariance | wavelet_HHL_gldm_GrayLevelVariance |
| wavelet_HHL_gldm_HighGrayLevelEmphasis | wavelet_HHL_gldm_HighGrayLevelEmphasis |
| **wavelet_HHL_gldm_LargeDependenceEmphasis** | wavelet_HHL_gldm_LargeDependenceEmphasis |
| wavelet_HHL_gldm_LargeDependenceHighGrayLevelEmphasis | wavelet_HHL_gldm_LargeDependenceHighGrayLevelEmphasis |
| wavelet_HHL_gldm_LargeDependenceLowGrayLevelEmphasis | wavelet_HHL_gldm_LargeDependenceLowGrayLevelEmphasis |
| wavelet_HHL_gldm_LowGrayLevelEmphasis | wavelet_HHL_gldm_LowGrayLevelEmphasis |
| wavelet_HHL_gldm_SmallDependenceEmphasis | wavelet_HHL_gldm_SmallDependenceEmphasis |
| wavelet_HHL_gldm_SmallDependenceHighGrayLevelEmphasis | wavelet_HHL_gldm_SmallDependenceHighGrayLevelEmphasis |
| **wavelet_HHL_gldm_SmallDependenceLowGrayLevelEmphasis** | wavelet_HHL_gldm_SmallDependenceLowGrayLevelEmphasis |
| wavelet_HHL_glrlm_GrayLevelNonUniformity | wavelet_HHL_glrlm_GrayLevelNonUniformity |
| wavelet_HHL_glrlm_GrayLevelNonUniformityNormalized | wavelet_HHL_glrlm_GrayLevelNonUniformityNormalized |
| wavelet_HHL_glrlm_GrayLevelVariance | wavelet_HHL_glrlm_GrayLevelVariance |
| wavelet_HHL_glrlm_HighGrayLevelRunEmphasis | wavelet_HHL_glrlm_HighGrayLevelRunEmphasis |
| **wavelet_HHL_glrlm_LongRunEmphasis** | wavelet_HHL_glrlm_LongRunEmphasis |
| wavelet_HHL_glrlm_LongRunHighGrayLevelEmphasis | wavelet_HHL_glrlm_LongRunHighGrayLevelEmphasis |
| wavelet_HHL_glrlm_LongRunLowGrayLevelEmphasis | wavelet_HHL_glrlm_LongRunLowGrayLevelEmphasis |
| wavelet_HHL_glrlm_LowGrayLevelRunEmphasis | wavelet_HHL_glrlm_LowGrayLevelRunEmphasis |
| **wavelet_HHL_glrlm_RunEntropy** | wavelet_HHL_glrlm_RunEntropy |
| wavelet_HHL_glrlm_RunLengthNonUniformity | wavelet_HHL_glrlm_RunLengthNonUniformity |
| **wavelet_HHL_glrlm_RunLengthNonUniformityNormalized** | wavelet_HHL_glrlm_RunLengthNonUniformityNormalized |
| **wavelet_HHL_glrlm_RunPercentage** | wavelet_HHL_glrlm_RunPercentage |
| **wavelet_HHL_glrlm_RunVariance** | wavelet_HHL_glrlm_RunVariance |
| **wavelet_HHL_glrlm_ShortRunEmphasis** | wavelet_HHL_glrlm_ShortRunEmphasis |
| wavelet_HHL_glrlm_ShortRunHighGrayLevelEmphasis | wavelet_HHL_glrlm_ShortRunHighGrayLevelEmphasis |
| wavelet_HHL_glrlm_ShortRunLowGrayLevelEmphasis | wavelet_HHL_glrlm_ShortRunLowGrayLevelEmphasis |
| wavelet_HHL_glszm_GrayLevelNonUniformity | wavelet_HHL_glszm_GrayLevelNonUniformity |
| wavelet_HHL_glszm_GrayLevelNonUniformityNormalized | wavelet_HHL_glszm_GrayLevelNonUniformityNormalized |
| wavelet_HHL_glszm_GrayLevelVariance | wavelet_HHL_glszm_GrayLevelVariance |
| wavelet_HHL_glszm_HighGrayLevelZoneEmphasis | wavelet_HHL_glszm_HighGrayLevelZoneEmphasis |
| wavelet_HHL_glszm_LargeAreaEmphasis | wavelet_HHL_glszm_LargeAreaEmphasis |
| wavelet_HHL_glszm_LargeAreaHighGrayLevelEmphasis | wavelet_HHL_glszm_LargeAreaHighGrayLevelEmphasis |
| wavelet_HHL_glszm_LargeAreaLowGrayLevelEmphasis | wavelet_HHL_glszm_LargeAreaLowGrayLevelEmphasis |
| wavelet_HHL_glszm_LowGrayLevelZoneEmphasis | wavelet_HHL_glszm_LowGrayLevelZoneEmphasis |
| wavelet_HHL_glszm_SizeZoneNonUniformity | wavelet_HHL_glszm_SizeZoneNonUniformity |
| wavelet_HHL_glszm_SizeZoneNonUniformityNormalized | wavelet_HHL_glszm_SizeZoneNonUniformityNormalized |
| **wavelet_HHL_glszm_SmallAreaEmphasis** | **wavelet_HHL_glszm_SmallAreaEmphasis** |
| wavelet_HHL_glszm_SmallAreaHighGrayLevelEmphasis | wavelet_HHL_glszm_SmallAreaHighGrayLevelEmphasis |
| wavelet_HHL_glszm_SmallAreaLowGrayLevelEmphasis | wavelet_HHL_glszm_SmallAreaLowGrayLevelEmphasis |
| **wavelet_HHL_glszm_ZoneEntropy** | wavelet_HHL_glszm_ZoneEntropy |
| wavelet_HHL_glszm_ZonePercentage | wavelet_HHL_glszm_ZonePercentage |
| wavelet_HHL_glszm_ZoneVariance | wavelet_HHL_glszm_ZoneVariance |
| wavelet_HHL_ngtdm_Busyness | wavelet_HHL_ngtdm_Busyness |
| **wavelet_HHL_ngtdm_Coarseness** | **wavelet_HHL_ngtdm_Coarseness** |
| wavelet_HHL_ngtdm_Complexity | wavelet_HHL_ngtdm_Complexity |
| **wavelet_HHL_ngtdm_Contrast** | **wavelet_HHL_ngtdm_Contrast** |
| wavelet_HHL_ngtdm_Strength | wavelet_HHL_ngtdm_Strength |
| wavelet_HLH_glcm_Autocorrelation | wavelet_HLH_glcm_Autocorrelation |
| wavelet_HLH_glcm_ClusterProminence | wavelet_HLH_glcm_ClusterProminence |
| wavelet_HLH_glcm_ClusterShade | wavelet_HLH_glcm_ClusterShade |
| wavelet_HLH_glcm_ClusterTendency | wavelet_HLH_glcm_ClusterTendency |
| wavelet_HLH_glcm_Contrast | wavelet_HLH_glcm_Contrast |
| wavelet_HLH_glcm_Correlation | wavelet_HLH_glcm_Correlation |
| wavelet_HLH_glcm_DifferenceAverage | wavelet_HLH_glcm_DifferenceAverage |
| wavelet_HLH_glcm_DifferenceEntropy | wavelet_HLH_glcm_DifferenceEntropy |
| wavelet_HLH_glcm_DifferenceVariance | wavelet_HLH_glcm_DifferenceVariance |
| wavelet_HLH_glcm_Id | wavelet_HLH_glcm_Id |
| wavelet_HLH_glcm_Idm | wavelet_HLH_glcm_Idm |
| wavelet_HLH_glcm_Idmn | wavelet_HLH_glcm_Idmn |
| wavelet_HLH_glcm_Idn | wavelet_HLH_glcm_Idn |
| wavelet_HLH_glcm_Imc1 | wavelet_HLH_glcm_Imc1 |
| wavelet_HLH_glcm_Imc2 | wavelet_HLH_glcm_Imc2 |
| wavelet_HLH_glcm_InverseVariance | wavelet_HLH_glcm_InverseVariance |
| wavelet_HLH_glcm_JointAverage | wavelet_HLH_glcm_JointAverage |
| wavelet_HLH_glcm_JointEnergy | wavelet_HLH_glcm_JointEnergy |
| wavelet_HLH_glcm_JointEntropy | wavelet_HLH_glcm_JointEntropy |
| wavelet_HLH_glcm_MaximumProbability | wavelet_HLH_glcm_MaximumProbability |
| wavelet_HLH_glcm_SumAverage | wavelet_HLH_glcm_SumAverage |
| wavelet_HLH_glcm_SumEntropy | wavelet_HLH_glcm_SumEntropy |
| wavelet_HLH_glcm_SumSquares | wavelet_HLH_glcm_SumSquares |
| wavelet_HLH_gldm_DependenceEntropy | wavelet_HLH_gldm_DependenceEntropy |
| wavelet_HLH_gldm_DependenceNonUniformity | wavelet_HLH_gldm_DependenceNonUniformity |
| wavelet_HLH_gldm_DependenceNonUniformityNormalized | wavelet_HLH_gldm_DependenceNonUniformityNormalized |
| wavelet_HLH_gldm_DependenceVariance | wavelet_HLH_gldm_DependenceVariance |
| wavelet_HLH_gldm_GrayLevelNonUniformity | wavelet_HLH_gldm_GrayLevelNonUniformity |
| wavelet_HLH_gldm_GrayLevelVariance | wavelet_HLH_gldm_GrayLevelVariance |
| wavelet_HLH_gldm_HighGrayLevelEmphasis | wavelet_HLH_gldm_HighGrayLevelEmphasis |
| **wavelet_HLH_gldm_LargeDependenceEmphasis** | wavelet_HLH_gldm_LargeDependenceEmphasis |
| wavelet_HLH_gldm_LargeDependenceHighGrayLevelEmphasis | wavelet_HLH_gldm_LargeDependenceHighGrayLevelEmphasis |
| wavelet_HLH_gldm_LargeDependenceLowGrayLevelEmphasis | wavelet_HLH_gldm_LargeDependenceLowGrayLevelEmphasis |
| wavelet_HLH_gldm_LowGrayLevelEmphasis | wavelet_HLH_gldm_LowGrayLevelEmphasis |
| **wavelet_HLH_gldm_SmallDependenceEmphasis** | wavelet_HLH_gldm_SmallDependenceEmphasis |
| wavelet_HLH_gldm_SmallDependenceHighGrayLevelEmphasis | wavelet_HLH_gldm_SmallDependenceHighGrayLevelEmphasis |
| **wavelet_HLH_gldm_SmallDependenceLowGrayLevelEmphasis** | wavelet_HLH_gldm_SmallDependenceLowGrayLevelEmphasis |
| wavelet_HLH_glrlm_GrayLevelNonUniformity | wavelet_HLH_glrlm_GrayLevelNonUniformity |
| wavelet_HLH_glrlm_GrayLevelNonUniformityNormalized | wavelet_HLH_glrlm_GrayLevelNonUniformityNormalized |
| wavelet_HLH_glrlm_GrayLevelVariance | wavelet_HLH_glrlm_GrayLevelVariance |
| wavelet_HLH_glrlm_HighGrayLevelRunEmphasis | wavelet_HLH_glrlm_HighGrayLevelRunEmphasis |
| **wavelet_HLH_glrlm_LongRunEmphasis** | wavelet_HLH_glrlm_LongRunEmphasis |
| wavelet_HLH_glrlm_LongRunHighGrayLevelEmphasis | wavelet_HLH_glrlm_LongRunHighGrayLevelEmphasis |
| wavelet_HLH_glrlm_LongRunLowGrayLevelEmphasis | wavelet_HLH_glrlm_LongRunLowGrayLevelEmphasis |
| wavelet_HLH_glrlm_LowGrayLevelRunEmphasis | wavelet_HLH_glrlm_LowGrayLevelRunEmphasis |
| wavelet_HLH_glrlm_RunEntropy | wavelet_HLH_glrlm_RunEntropy |
| wavelet_HLH_glrlm_RunLengthNonUniformity | wavelet_HLH_glrlm_RunLengthNonUniformity |
| **wavelet_HLH_glrlm_RunLengthNonUniformityNormalized** | wavelet_HLH_glrlm_RunLengthNonUniformityNormalized |
| **wavelet_HLH_glrlm_RunPercentage** | wavelet_HLH_glrlm_RunPercentage |
| **wavelet_HLH_glrlm_RunVariance** | wavelet_HLH_glrlm_RunVariance |
| **wavelet_HLH_glrlm_ShortRunEmphasis** | wavelet_HLH_glrlm_ShortRunEmphasis |
| wavelet_HLH_glrlm_ShortRunHighGrayLevelEmphasis | wavelet_HLH_glrlm_ShortRunHighGrayLevelEmphasis |
| **wavelet_HLH_glrlm_ShortRunLowGrayLevelEmphasis** | wavelet_HLH_glrlm_ShortRunLowGrayLevelEmphasis |
| wavelet_HLH_glszm_GrayLevelNonUniformity | wavelet_HLH_glszm_GrayLevelNonUniformity |
| wavelet_HLH_glszm_GrayLevelNonUniformityNormalized | wavelet_HLH_glszm_GrayLevelNonUniformityNormalized |
| wavelet_HLH_glszm_GrayLevelVariance | wavelet_HLH_glszm_GrayLevelVariance |
| wavelet_HLH_glszm_HighGrayLevelZoneEmphasis | wavelet_HLH_glszm_HighGrayLevelZoneEmphasis |
| wavelet_HLH_glszm_LargeAreaEmphasis | wavelet_HLH_glszm_LargeAreaEmphasis |
| wavelet_HLH_glszm_LargeAreaHighGrayLevelEmphasis | wavelet_HLH_glszm_LargeAreaHighGrayLevelEmphasis |
| wavelet_HLH_glszm_LargeAreaLowGrayLevelEmphasis | wavelet_HLH_glszm_LargeAreaLowGrayLevelEmphasis |
| wavelet_HLH_glszm_LowGrayLevelZoneEmphasis | wavelet_HLH_glszm_LowGrayLevelZoneEmphasis |
| wavelet_HLH_glszm_SizeZoneNonUniformity | wavelet_HLH_glszm_SizeZoneNonUniformity |
| wavelet_HLH_glszm_SizeZoneNonUniformityNormalized | wavelet_HLH_glszm_SizeZoneNonUniformityNormalized |
| **wavelet_HLH_glszm_SmallAreaEmphasis** | wavelet_HLH_glszm_SmallAreaEmphasis |
| **wavelet_HLH_glszm_SmallAreaHighGrayLevelEmphasis** | wavelet_HLH_glszm_SmallAreaHighGrayLevelEmphasis |
| wavelet_HLH_glszm_SmallAreaLowGrayLevelEmphasis | wavelet_HLH_glszm_SmallAreaLowGrayLevelEmphasis |
| **wavelet_HLH_glszm_ZoneEntropy** | wavelet_HLH_glszm_ZoneEntropy |
| **wavelet_HLH_glszm_ZonePercentage** | wavelet_HLH_glszm_ZonePercentage |
| wavelet_HLH_glszm_ZoneVariance | wavelet_HLH_glszm_ZoneVariance |
| wavelet_HLH_ngtdm_Busyness | wavelet_HLH_ngtdm_Busyness |
| **wavelet_HLH_ngtdm_Coarseness** | **wavelet_HLH_ngtdm_Coarseness** |
| **wavelet_HLH_ngtdm_Complexity** | wavelet_HLH_ngtdm_Complexity |
| **wavelet_HLH_ngtdm_Contrast** | **wavelet_HLH_ngtdm_Contrast** |
| wavelet_HLH_ngtdm_Strength | wavelet_HLH_ngtdm_Strength |
| wavelet_HLL_glcm_Autocorrelation | wavelet_HLL_glcm_Autocorrelation |
| wavelet_HLL_glcm_ClusterProminence | wavelet_HLL_glcm_ClusterProminence |
| wavelet_HLL_glcm_ClusterShade | wavelet_HLL_glcm_ClusterShade |
| wavelet_HLL_glcm_ClusterTendency | wavelet_HLL_glcm_ClusterTendency |
| wavelet_HLL_glcm_Contrast | wavelet_HLL_glcm_Contrast |
| wavelet_HLL_glcm_Correlation | wavelet_HLL_glcm_Correlation |
| wavelet_HLL_glcm_DifferenceAverage | wavelet_HLL_glcm_DifferenceAverage |
| wavelet_HLL_glcm_DifferenceEntropy | wavelet_HLL_glcm_DifferenceEntropy |
| wavelet_HLL_glcm_DifferenceVariance | wavelet_HLL_glcm_DifferenceVariance |
| wavelet_HLL_glcm_Id | wavelet_HLL_glcm_Id |
| wavelet_HLL_glcm_Idm | wavelet_HLL_glcm_Idm |
| **wavelet_HLL_glcm_Idmn** | wavelet_HLL_glcm_Idmn |
| **wavelet_HLL_glcm_Idn** | wavelet_HLL_glcm_Idn |
| wavelet_HLL_glcm_Imc1 | wavelet_HLL_glcm_Imc1 |
| wavelet_HLL_glcm_Imc2 | wavelet_HLL_glcm_Imc2 |
| wavelet_HLL_glcm_InverseVariance | wavelet_HLL_glcm_InverseVariance |
| wavelet_HLL_glcm_JointAverage | wavelet_HLL_glcm_JointAverage |
| wavelet_HLL_glcm_JointEnergy | wavelet_HLL_glcm_JointEnergy |
| wavelet_HLL_glcm_JointEntropy | wavelet_HLL_glcm_JointEntropy |
| wavelet_HLL_glcm_MaximumProbability | wavelet_HLL_glcm_MaximumProbability |
| wavelet_HLL_glcm_SumAverage | wavelet_HLL_glcm_SumAverage |
| wavelet_HLL_glcm_SumEntropy | wavelet_HLL_glcm_SumEntropy |
| wavelet_HLL_glcm_SumSquares | wavelet_HLL_glcm_SumSquares |
| wavelet_HLL_gldm_DependenceEntropy | wavelet_HLL_gldm_DependenceEntropy |
| wavelet_HLL_gldm_DependenceNonUniformity | wavelet_HLL_gldm_DependenceNonUniformity |
| wavelet_HLL_gldm_DependenceNonUniformityNormalized | wavelet_HLL_gldm_DependenceNonUniformityNormalized |
| wavelet_HLL_gldm_DependenceVariance | wavelet_HLL_gldm_DependenceVariance |
| wavelet_HLL_gldm_GrayLevelNonUniformity | wavelet_HLL_gldm_GrayLevelNonUniformity |
| wavelet_HLL_gldm_GrayLevelVariance | wavelet_HLL_gldm_GrayLevelVariance |
| wavelet_HLL_gldm_HighGrayLevelEmphasis | wavelet_HLL_gldm_HighGrayLevelEmphasis |
| wavelet_HLL_gldm_LargeDependenceEmphasis | wavelet_HLL_gldm_LargeDependenceEmphasis |
| wavelet_HLL_gldm_LargeDependenceHighGrayLevelEmphasis | wavelet_HLL_gldm_LargeDependenceHighGrayLevelEmphasis |
| **wavelet_HLL_gldm_LargeDependenceLowGrayLevelEmphasis** | wavelet_HLL_gldm_LargeDependenceLowGrayLevelEmphasis |
| **wavelet_HLL_gldm_LowGrayLevelEmphasis** | wavelet_HLL_gldm_LowGrayLevelEmphasis |
| wavelet_HLL_gldm_SmallDependenceEmphasis | wavelet_HLL_gldm_SmallDependenceEmphasis |
| wavelet_HLL_gldm_SmallDependenceHighGrayLevelEmphasis | wavelet_HLL_gldm_SmallDependenceHighGrayLevelEmphasis |
| **wavelet_HLL_gldm_SmallDependenceLowGrayLevelEmphasis** | wavelet_HLL_gldm_SmallDependenceLowGrayLevelEmphasis |
| wavelet_HLL_glrlm_GrayLevelNonUniformity | wavelet_HLL_glrlm_GrayLevelNonUniformity |
| wavelet_HLL_glrlm_GrayLevelNonUniformityNormalized | wavelet_HLL_glrlm_GrayLevelNonUniformityNormalized |
| wavelet_HLL_glrlm_GrayLevelVariance | wavelet_HLL_glrlm_GrayLevelVariance |
| wavelet_HLL_glrlm_HighGrayLevelRunEmphasis | wavelet_HLL_glrlm_HighGrayLevelRunEmphasis |
| wavelet_HLL_glrlm_LongRunEmphasis | wavelet_HLL_glrlm_LongRunEmphasis |
| wavelet_HLL_glrlm_LongRunHighGrayLevelEmphasis | wavelet_HLL_glrlm_LongRunHighGrayLevelEmphasis |
| **wavelet_HLL_glrlm_LongRunLowGrayLevelEmphasis** | wavelet_HLL_glrlm_LongRunLowGrayLevelEmphasis |
| **wavelet_HLL_glrlm_LowGrayLevelRunEmphasis** | wavelet_HLL_glrlm_LowGrayLevelRunEmphasis |
| wavelet_HLL_glrlm_RunEntropy | wavelet_HLL_glrlm_RunEntropy |
| wavelet_HLL_glrlm_RunLengthNonUniformity | wavelet_HLL_glrlm_RunLengthNonUniformity |
| wavelet_HLL_glrlm_RunLengthNonUniformityNormalized | wavelet_HLL_glrlm_RunLengthNonUniformityNormalized |
| wavelet_HLL_glrlm_RunPercentage | wavelet_HLL_glrlm_RunPercentage |
| wavelet_HLL_glrlm_RunVariance | wavelet_HLL_glrlm_RunVariance |
| wavelet_HLL_glrlm_ShortRunEmphasis | wavelet_HLL_glrlm_ShortRunEmphasis |
| wavelet_HLL_glrlm_ShortRunHighGrayLevelEmphasis | wavelet_HLL_glrlm_ShortRunHighGrayLevelEmphasis |
| **wavelet_HLL_glrlm_ShortRunLowGrayLevelEmphasis** | wavelet_HLL_glrlm_ShortRunLowGrayLevelEmphasis |
| wavelet_HLL_glszm_GrayLevelNonUniformity | wavelet_HLL_glszm_GrayLevelNonUniformity |
| wavelet_HLL_glszm_GrayLevelNonUniformityNormalized | wavelet_HLL_glszm_GrayLevelNonUniformityNormalized |
| wavelet_HLL_glszm_GrayLevelVariance | wavelet_HLL_glszm_GrayLevelVariance |
| wavelet_HLL_glszm_HighGrayLevelZoneEmphasis | wavelet_HLL_glszm_HighGrayLevelZoneEmphasis |
| wavelet_HLL_glszm_LargeAreaEmphasis | wavelet_HLL_glszm_LargeAreaEmphasis |
| wavelet_HLL_glszm_LargeAreaHighGrayLevelEmphasis | wavelet_HLL_glszm_LargeAreaHighGrayLevelEmphasis |
| wavelet_HLL_glszm_LargeAreaLowGrayLevelEmphasis | wavelet_HLL_glszm_LargeAreaLowGrayLevelEmphasis |
| **wavelet_HLL_glszm_LowGrayLevelZoneEmphasis** | wavelet_HLL_glszm_LowGrayLevelZoneEmphasis |
| wavelet_HLL_glszm_SizeZoneNonUniformity | wavelet_HLL_glszm_SizeZoneNonUniformity |
| wavelet_HLL_glszm_SizeZoneNonUniformityNormalized | wavelet_HLL_glszm_SizeZoneNonUniformityNormalized |
| wavelet_HLL_glszm_SmallAreaEmphasis | wavelet_HLL_glszm_SmallAreaEmphasis |
| wavelet_HLL_glszm_SmallAreaHighGrayLevelEmphasis | wavelet_HLL_glszm_SmallAreaHighGrayLevelEmphasis |
| wavelet_HLL_glszm_SmallAreaLowGrayLevelEmphasis | wavelet_HLL_glszm_SmallAreaLowGrayLevelEmphasis |
| **wavelet_HLL_glszm_ZoneEntropy** | wavelet_HLL_glszm_ZoneEntropy |
| wavelet_HLL_glszm_ZonePercentage | wavelet_HLL_glszm_ZonePercentage |
| wavelet_HLL_glszm_ZoneVariance | wavelet_HLL_glszm_ZoneVariance |
| wavelet_HLL_ngtdm_Busyness | wavelet_HLL_ngtdm_Busyness |
| **wavelet_HLL_ngtdm_Coarseness** | **wavelet_HLL_ngtdm_Coarseness** |
| wavelet_HLL_ngtdm_Complexity | wavelet_HLL_ngtdm_Complexity |
| **wavelet_HLL_ngtdm_Contrast** | wavelet_HLL_ngtdm_Contrast |
| wavelet_HLL_ngtdm_Strength | wavelet_HLL_ngtdm_Strength |
| wavelet_LHH_glcm_Autocorrelation | wavelet_LHH_glcm_Autocorrelation |
| wavelet_LHH_glcm_ClusterProminence | wavelet_LHH_glcm_ClusterProminence |
| wavelet_LHH_glcm_ClusterShade | wavelet_LHH_glcm_ClusterShade |
| wavelet_LHH_glcm_ClusterTendency | wavelet_LHH_glcm_ClusterTendency |
| wavelet_LHH_glcm_Contrast | wavelet_LHH_glcm_Contrast |
| wavelet_LHH_glcm_Correlation | wavelet_LHH_glcm_Correlation |
| wavelet_LHH_glcm_DifferenceAverage | wavelet_LHH_glcm_DifferenceAverage |
| wavelet_LHH_glcm_DifferenceEntropy | wavelet_LHH_glcm_DifferenceEntropy |
| wavelet_LHH_glcm_DifferenceVariance | wavelet_LHH_glcm_DifferenceVariance |
| wavelet_LHH_glcm_Id | wavelet_LHH_glcm_Id |
| wavelet_LHH_glcm_Idm | wavelet_LHH_glcm_Idm |
| wavelet_LHH_glcm_Idmn | wavelet_LHH_glcm_Idmn |
| wavelet_LHH_glcm_Idn | wavelet_LHH_glcm_Idn |
| wavelet_LHH_glcm_Imc1 | wavelet_LHH_glcm_Imc1 |
| wavelet_LHH_glcm_Imc2 | wavelet_LHH_glcm_Imc2 |
| wavelet_LHH_glcm_InverseVariance | wavelet_LHH_glcm_InverseVariance |
| wavelet_LHH_glcm_JointAverage | wavelet_LHH_glcm_JointAverage |
| wavelet_LHH_glcm_JointEnergy | wavelet_LHH_glcm_JointEnergy |
| wavelet_LHH_glcm_JointEntropy | wavelet_LHH_glcm_JointEntropy |
| wavelet_LHH_glcm_MaximumProbability | wavelet_LHH_glcm_MaximumProbability |
| wavelet_LHH_glcm_SumAverage | wavelet_LHH_glcm_SumAverage |
| wavelet_LHH_glcm_SumEntropy | wavelet_LHH_glcm_SumEntropy |
| wavelet_LHH_glcm_SumSquares | wavelet_LHH_glcm_SumSquares |
| wavelet_LHH_gldm_DependenceEntropy | wavelet_LHH_gldm_DependenceEntropy |
| wavelet_LHH_gldm_DependenceNonUniformity | wavelet_LHH_gldm_DependenceNonUniformity |
| wavelet_LHH_gldm_DependenceNonUniformityNormalized | wavelet_LHH_gldm_DependenceNonUniformityNormalized |
| wavelet_LHH_gldm_DependenceVariance | wavelet_LHH_gldm_DependenceVariance |
| wavelet_LHH_gldm_GrayLevelNonUniformity | wavelet_LHH_gldm_GrayLevelNonUniformity |
| wavelet_LHH_gldm_GrayLevelVariance | wavelet_LHH_gldm_GrayLevelVariance |
| wavelet_LHH_gldm_HighGrayLevelEmphasis | wavelet_LHH_gldm_HighGrayLevelEmphasis |
| **wavelet_LHH_gldm_LargeDependenceEmphasis** | wavelet_LHH_gldm_LargeDependenceEmphasis |
| wavelet_LHH_gldm_LargeDependenceHighGrayLevelEmphasis | wavelet_LHH_gldm_LargeDependenceHighGrayLevelEmphasis |
| wavelet_LHH_gldm_LargeDependenceLowGrayLevelEmphasis | wavelet_LHH_gldm_LargeDependenceLowGrayLevelEmphasis |
| wavelet_LHH_gldm_LowGrayLevelEmphasis | wavelet_LHH_gldm_LowGrayLevelEmphasis |
| **wavelet_LHH_gldm_SmallDependenceEmphasis** | wavelet_LHH_gldm_SmallDependenceEmphasis |
| wavelet_LHH_gldm_SmallDependenceHighGrayLevelEmphasis | wavelet_LHH_gldm_SmallDependenceHighGrayLevelEmphasis |
| **wavelet_LHH_gldm_SmallDependenceLowGrayLevelEmphasis** | wavelet_LHH_gldm_SmallDependenceLowGrayLevelEmphasis |
| wavelet_LHH_glrlm_GrayLevelNonUniformity | wavelet_LHH_glrlm_GrayLevelNonUniformity |
| wavelet_LHH_glrlm_GrayLevelNonUniformityNormalized | wavelet_LHH_glrlm_GrayLevelNonUniformityNormalized |
| wavelet_LHH_glrlm_GrayLevelVariance | wavelet_LHH_glrlm_GrayLevelVariance |
| wavelet_LHH_glrlm_HighGrayLevelRunEmphasis | wavelet_LHH_glrlm_HighGrayLevelRunEmphasis |
| **wavelet_LHH_glrlm_LongRunEmphasis** | wavelet_LHH_glrlm_LongRunEmphasis |
| wavelet_LHH_glrlm_LongRunHighGrayLevelEmphasis | wavelet_LHH_glrlm_LongRunHighGrayLevelEmphasis |
| wavelet_LHH_glrlm_LongRunLowGrayLevelEmphasis | wavelet_LHH_glrlm_LongRunLowGrayLevelEmphasis |
| wavelet_LHH_glrlm_LowGrayLevelRunEmphasis | wavelet_LHH_glrlm_LowGrayLevelRunEmphasis |
| **wavelet_LHH_glrlm_RunEntropy** | wavelet_LHH_glrlm_RunEntropy |
| wavelet_LHH_glrlm_RunLengthNonUniformity | wavelet_LHH_glrlm_RunLengthNonUniformity |
| **wavelet_LHH_glrlm_RunLengthNonUniformityNormalized** | wavelet_LHH_glrlm_RunLengthNonUniformityNormalized |
| **wavelet_LHH_glrlm_RunPercentage** | wavelet_LHH_glrlm_RunPercentage |
| **wavelet_LHH_glrlm_RunVariance** | wavelet_LHH_glrlm_RunVariance |
| **wavelet_LHH_glrlm_ShortRunEmphasis** | wavelet_LHH_glrlm_ShortRunEmphasis |
| wavelet_LHH_glrlm_ShortRunHighGrayLevelEmphasis | wavelet_LHH_glrlm_ShortRunHighGrayLevelEmphasis |
| wavelet_LHH_glrlm_ShortRunLowGrayLevelEmphasis | wavelet_LHH_glrlm_ShortRunLowGrayLevelEmphasis |
| wavelet_LHH_glszm_GrayLevelNonUniformity | wavelet_LHH_glszm_GrayLevelNonUniformity |
| wavelet_LHH_glszm_GrayLevelNonUniformityNormalized | wavelet_LHH_glszm_GrayLevelNonUniformityNormalized |
| wavelet_LHH_glszm_GrayLevelVariance | wavelet_LHH_glszm_GrayLevelVariance |
| wavelet_LHH_glszm_HighGrayLevelZoneEmphasis | wavelet_LHH_glszm_HighGrayLevelZoneEmphasis |
| wavelet_LHH_glszm_LargeAreaEmphasis | wavelet_LHH_glszm_LargeAreaEmphasis |
| wavelet_LHH_glszm_LargeAreaHighGrayLevelEmphasis | wavelet_LHH_glszm_LargeAreaHighGrayLevelEmphasis |
| wavelet_LHH_glszm_LargeAreaLowGrayLevelEmphasis | wavelet_LHH_glszm_LargeAreaLowGrayLevelEmphasis |
| wavelet_LHH_glszm_LowGrayLevelZoneEmphasis | wavelet_LHH_glszm_LowGrayLevelZoneEmphasis |
| wavelet_LHH_glszm_SizeZoneNonUniformity | wavelet_LHH_glszm_SizeZoneNonUniformity |
| **wavelet_LHH_glszm_SizeZoneNonUniformityNormalized** | wavelet_LHH_glszm_SizeZoneNonUniformityNormalized |
| **wavelet_LHH_glszm_SmallAreaEmphasis** | wavelet_LHH_glszm_SmallAreaEmphasis |
| **wavelet_LHH_glszm_SmallAreaHighGrayLevelEmphasis** | wavelet_LHH_glszm_SmallAreaHighGrayLevelEmphasis |
| wavelet_LHH_glszm_SmallAreaLowGrayLevelEmphasis | wavelet_LHH_glszm_SmallAreaLowGrayLevelEmphasis |
| **wavelet_LHH_glszm_ZoneEntropy** | **wavelet_LHH_glszm_ZoneEntropy** |
| **wavelet_LHH_glszm_ZonePercentage** | wavelet_LHH_glszm_ZonePercentage |
| wavelet_LHH_glszm_ZoneVariance | wavelet_LHH_glszm_ZoneVariance |
| wavelet_LHH_ngtdm_Busyness | wavelet_LHH_ngtdm_Busyness |
| **wavelet_LHH_ngtdm_Coarseness** | **wavelet_LHH_ngtdm_Coarseness** |
| wavelet_LHH_ngtdm_Complexity | wavelet_LHH_ngtdm_Complexity |
| **wavelet_LHH_ngtdm_Contrast** | **wavelet_LHH_ngtdm_Contrast** |
| wavelet_LHH_ngtdm_Strength | wavelet_LHH_ngtdm_Strength |
| wavelet_LHL_glcm_Autocorrelation | wavelet_LHL_glcm_Autocorrelation |
| wavelet_LHL_glcm_ClusterProminence | wavelet_LHL_glcm_ClusterProminence |
| wavelet_LHL_glcm_ClusterShade | wavelet_LHL_glcm_ClusterShade |
| wavelet_LHL_glcm_ClusterTendency | wavelet_LHL_glcm_ClusterTendency |
| wavelet_LHL_glcm_Contrast | wavelet_LHL_glcm_Contrast |
| wavelet_LHL_glcm_Correlation | wavelet_LHL_glcm_Correlation |
| wavelet_LHL_glcm_DifferenceAverage | wavelet_LHL_glcm_DifferenceAverage |
| wavelet_LHL_glcm_DifferenceEntropy | wavelet_LHL_glcm_DifferenceEntropy |
| wavelet_LHL_glcm_DifferenceVariance | wavelet_LHL_glcm_DifferenceVariance |
| wavelet_LHL_glcm_Id | wavelet_LHL_glcm_Id |
| wavelet_LHL_glcm_Idm | wavelet_LHL_glcm_Idm |
| **wavelet_LHL_glcm_Idmn** | wavelet_LHL_glcm_Idmn |
| **wavelet_LHL_glcm_Idn** | wavelet_LHL_glcm_Idn |
| wavelet_LHL_glcm_Imc1 | wavelet_LHL_glcm_Imc1 |
| wavelet_LHL_glcm_Imc2 | wavelet_LHL_glcm_Imc2 |
| wavelet_LHL_glcm_InverseVariance | wavelet_LHL_glcm_InverseVariance |
| wavelet_LHL_glcm_JointAverage | wavelet_LHL_glcm_JointAverage |
| wavelet_LHL_glcm_JointEnergy | wavelet_LHL_glcm_JointEnergy |
| wavelet_LHL_glcm_JointEntropy | wavelet_LHL_glcm_JointEntropy |
| **wavelet_LHL_glcm_MaximumProbability** | wavelet_LHL_glcm_MaximumProbability |
| wavelet_LHL_glcm_SumAverage | wavelet_LHL_glcm_SumAverage |
| wavelet_LHL_glcm_SumEntropy | wavelet_LHL_glcm_SumEntropy |
| wavelet_LHL_glcm_SumSquares | wavelet_LHL_glcm_SumSquares |
| wavelet_LHL_gldm_DependenceEntropy | wavelet_LHL_gldm_DependenceEntropy |
| wavelet_LHL_gldm_DependenceNonUniformity | wavelet_LHL_gldm_DependenceNonUniformity |
| **wavelet_LHL_gldm_DependenceNonUniformityNormalized** | wavelet_LHL_gldm_DependenceNonUniformityNormalized |
| wavelet_LHL_gldm_DependenceVariance | wavelet_LHL_gldm_DependenceVariance |
| wavelet_LHL_gldm_GrayLevelNonUniformity | wavelet_LHL_gldm_GrayLevelNonUniformity |
| wavelet_LHL_gldm_GrayLevelVariance | wavelet_LHL_gldm_GrayLevelVariance |
| wavelet_LHL_gldm_HighGrayLevelEmphasis | wavelet_LHL_gldm_HighGrayLevelEmphasis |
| wavelet_LHL_gldm_LargeDependenceEmphasis | wavelet_LHL_gldm_LargeDependenceEmphasis |
| wavelet_LHL_gldm_LargeDependenceHighGrayLevelEmphasis | wavelet_LHL_gldm_LargeDependenceHighGrayLevelEmphasis |
| wavelet_LHL_gldm_LargeDependenceLowGrayLevelEmphasis | wavelet_LHL_gldm_LargeDependenceLowGrayLevelEmphasis |
| **wavelet_LHL_gldm_LowGrayLevelEmphasis** | wavelet_LHL_gldm_LowGrayLevelEmphasis |
| wavelet_LHL_gldm_SmallDependenceEmphasis | wavelet_LHL_gldm_SmallDependenceEmphasis |
| wavelet_LHL_gldm_SmallDependenceHighGrayLevelEmphasis | wavelet_LHL_gldm_SmallDependenceHighGrayLevelEmphasis |
| **wavelet_LHL_gldm_SmallDependenceLowGrayLevelEmphasis** | **wavelet_LHL_gldm_SmallDependenceLowGrayLevelEmphasis** |
| wavelet_LHL_glrlm_GrayLevelNonUniformity | wavelet_LHL_glrlm_GrayLevelNonUniformity |
| wavelet_LHL_glrlm_GrayLevelNonUniformityNormalized | wavelet_LHL_glrlm_GrayLevelNonUniformityNormalized |
| wavelet_LHL_glrlm_GrayLevelVariance | wavelet_LHL_glrlm_GrayLevelVariance |
| wavelet_LHL_glrlm_HighGrayLevelRunEmphasis | wavelet_LHL_glrlm_HighGrayLevelRunEmphasis |
| wavelet_LHL_glrlm_LongRunEmphasis | wavelet_LHL_glrlm_LongRunEmphasis |
| wavelet_LHL_glrlm_LongRunHighGrayLevelEmphasis | wavelet_LHL_glrlm_LongRunHighGrayLevelEmphasis |
| wavelet_LHL_glrlm_LongRunLowGrayLevelEmphasis | wavelet_LHL_glrlm_LongRunLowGrayLevelEmphasis |
| **wavelet_LHL_glrlm_LowGrayLevelRunEmphasis** | wavelet_LHL_glrlm_LowGrayLevelRunEmphasis |
| **wavelet_LHL_glrlm_RunEntropy** | wavelet_LHL_glrlm_RunEntropy |
| wavelet_LHL_glrlm_RunLengthNonUniformity | wavelet_LHL_glrlm_RunLengthNonUniformity |
| **wavelet_LHL_glrlm_RunLengthNonUniformityNormalized** | wavelet_LHL_glrlm_RunLengthNonUniformityNormalized |
| **wavelet_LHL_glrlm_RunPercentage** | wavelet_LHL_glrlm_RunPercentage |
| wavelet_LHL_glrlm_RunVariance | wavelet_LHL_glrlm_RunVariance |
| wavelet_LHL_glrlm_ShortRunEmphasis | wavelet_LHL_glrlm_ShortRunEmphasis |
| wavelet_LHL_glrlm_ShortRunHighGrayLevelEmphasis | wavelet_LHL_glrlm_ShortRunHighGrayLevelEmphasis |
| **wavelet_LHL_glrlm_ShortRunLowGrayLevelEmphasis** | wavelet_LHL_glrlm_ShortRunLowGrayLevelEmphasis |
| wavelet_LHL_glszm_GrayLevelNonUniformity | wavelet_LHL_glszm_GrayLevelNonUniformity |
| wavelet_LHL_glszm_GrayLevelNonUniformityNormalized | wavelet_LHL_glszm_GrayLevelNonUniformityNormalized |
| wavelet_LHL_glszm_GrayLevelVariance | wavelet_LHL_glszm_GrayLevelVariance |
| wavelet_LHL_glszm_HighGrayLevelZoneEmphasis | wavelet_LHL_glszm_HighGrayLevelZoneEmphasis |
| wavelet_LHL_glszm_LargeAreaEmphasis | wavelet_LHL_glszm_LargeAreaEmphasis |
| wavelet_LHL_glszm_LargeAreaHighGrayLevelEmphasis | wavelet_LHL_glszm_LargeAreaHighGrayLevelEmphasis |
| wavelet_LHL_glszm_LargeAreaLowGrayLevelEmphasis | wavelet_LHL_glszm_LargeAreaLowGrayLevelEmphasis |
| **wavelet_LHL_glszm_LowGrayLevelZoneEmphasis** | wavelet_LHL_glszm_LowGrayLevelZoneEmphasis |
| wavelet_LHL_glszm_SizeZoneNonUniformity | wavelet_LHL_glszm_SizeZoneNonUniformity |
| wavelet_LHL_glszm_SizeZoneNonUniformityNormalized | wavelet_LHL_glszm_SizeZoneNonUniformityNormalized |
| **wavelet_LHL_glszm_SmallAreaEmphasis** | wavelet_LHL_glszm_SmallAreaEmphasis |
| wavelet_LHL_glszm_SmallAreaHighGrayLevelEmphasis | wavelet_LHL_glszm_SmallAreaHighGrayLevelEmphasis |
| wavelet_LHL_glszm_SmallAreaLowGrayLevelEmphasis | wavelet_LHL_glszm_SmallAreaLowGrayLevelEmphasis |
| **wavelet_LHL_glszm_ZoneEntropy** | wavelet_LHL_glszm_ZoneEntropy |
| wavelet_LHL_glszm_ZonePercentage | wavelet_LHL_glszm_ZonePercentage |
| wavelet_LHL_glszm_ZoneVariance | wavelet_LHL_glszm_ZoneVariance |
| wavelet_LHL_ngtdm_Busyness | wavelet_LHL_ngtdm_Busyness |
| **wavelet_LHL_ngtdm_Coarseness** | **wavelet_LHL_ngtdm_Coarseness** |
| **wavelet_LHL_ngtdm_Complexity** | wavelet_LHL_ngtdm_Complexity |
| **wavelet_LHL_ngtdm_Contrast** | wavelet_LHL_ngtdm_Contrast |
| wavelet_LHL_ngtdm_Strength | wavelet_LHL_ngtdm_Strength |
| wavelet_LLH_glcm_Autocorrelation | wavelet_LLH_glcm_Autocorrelation |
| wavelet_LLH_glcm_ClusterProminence | wavelet_LLH_glcm_ClusterProminence |
| wavelet_LLH_glcm_ClusterShade | wavelet_LLH_glcm_ClusterShade |
| wavelet_LLH_glcm_ClusterTendency | wavelet_LLH_glcm_ClusterTendency |
| wavelet_LLH_glcm_Contrast | wavelet_LLH_glcm_Contrast |
| wavelet_LLH_glcm_Correlation | wavelet_LLH_glcm_Correlation |
| wavelet_LLH_glcm_DifferenceAverage | wavelet_LLH_glcm_DifferenceAverage |
| wavelet_LLH_glcm_DifferenceEntropy | wavelet_LLH_glcm_DifferenceEntropy |
| wavelet_LLH_glcm_DifferenceVariance | wavelet_LLH_glcm_DifferenceVariance |
| wavelet_LLH_glcm_Id | wavelet_LLH_glcm_Id |
| wavelet_LLH_glcm_Idm | wavelet_LLH_glcm_Idm |
| wavelet_LLH_glcm_Idmn | wavelet_LLH_glcm_Idmn |
| wavelet_LLH_glcm_Idn | wavelet_LLH_glcm_Idn |
| **wavelet_LLH_glcm_Imc1** | wavelet_LLH_glcm_Imc1 |
| wavelet_LLH_glcm_Imc2 | wavelet_LLH_glcm_Imc2 |
| wavelet_LLH_glcm_InverseVariance | wavelet_LLH_glcm_InverseVariance |
| wavelet_LLH_glcm_JointAverage | wavelet_LLH_glcm_JointAverage |
| wavelet_LLH_glcm_JointEnergy | wavelet_LLH_glcm_JointEnergy |
| wavelet_LLH_glcm_JointEntropy | wavelet_LLH_glcm_JointEntropy |
| wavelet_LLH_glcm_MaximumProbability | wavelet_LLH_glcm_MaximumProbability |
| wavelet_LLH_glcm_SumAverage | wavelet_LLH_glcm_SumAverage |
| wavelet_LLH_glcm_SumEntropy | wavelet_LLH_glcm_SumEntropy |
| wavelet_LLH_glcm_SumSquares | wavelet_LLH_glcm_SumSquares |
| **wavelet_LLH_gldm_DependenceEntropy** | wavelet_LLH_gldm_DependenceEntropy |
| wavelet_LLH_gldm_DependenceNonUniformity | wavelet_LLH_gldm_DependenceNonUniformity |
| **wavelet_LLH_gldm_DependenceNonUniformityNormalized** | wavelet_LLH_gldm_DependenceNonUniformityNormalized |
| **wavelet_LLH_gldm_DependenceVariance** | wavelet_LLH_gldm_DependenceVariance |
| wavelet_LLH_gldm_GrayLevelNonUniformity | wavelet_LLH_gldm_GrayLevelNonUniformity |
| wavelet_LLH_gldm_GrayLevelVariance | wavelet_LLH_gldm_GrayLevelVariance |
| wavelet_LLH_gldm_HighGrayLevelEmphasis | wavelet_LLH_gldm_HighGrayLevelEmphasis |
| wavelet_LLH_gldm_LargeDependenceEmphasis | wavelet_LLH_gldm_LargeDependenceEmphasis |
| wavelet_LLH_gldm_LargeDependenceHighGrayLevelEmphasis | wavelet_LLH_gldm_LargeDependenceHighGrayLevelEmphasis |
| wavelet_LLH_gldm_LargeDependenceLowGrayLevelEmphasis | wavelet_LLH_gldm_LargeDependenceLowGrayLevelEmphasis |
| wavelet_LLH_gldm_LowGrayLevelEmphasis | wavelet_LLH_gldm_LowGrayLevelEmphasis |
| **wavelet_LLH_gldm_SmallDependenceEmphasis** | **wavelet_LLH_gldm_SmallDependenceEmphasis** |
| wavelet_LLH_gldm_SmallDependenceHighGrayLevelEmphasis | wavelet_LLH_gldm_SmallDependenceHighGrayLevelEmphasis |
| **wavelet_LLH_gldm_SmallDependenceLowGrayLevelEmphasis** | wavelet_LLH_gldm_SmallDependenceLowGrayLevelEmphasis |
| wavelet_LLH_glrlm_GrayLevelNonUniformity | wavelet_LLH_glrlm_GrayLevelNonUniformity |
| wavelet_LLH_glrlm_GrayLevelNonUniformityNormalized | wavelet_LLH_glrlm_GrayLevelNonUniformityNormalized |
| wavelet_LLH_glrlm_GrayLevelVariance | wavelet_LLH_glrlm_GrayLevelVariance |
| wavelet_LLH_glrlm_HighGrayLevelRunEmphasis | wavelet_LLH_glrlm_HighGrayLevelRunEmphasis |
| wavelet_LLH_glrlm_LongRunEmphasis | wavelet_LLH_glrlm_LongRunEmphasis |
| wavelet_LLH_glrlm_LongRunHighGrayLevelEmphasis | wavelet_LLH_glrlm_LongRunHighGrayLevelEmphasis |
| wavelet_LLH_glrlm_LongRunLowGrayLevelEmphasis | wavelet_LLH_glrlm_LongRunLowGrayLevelEmphasis |
| wavelet_LLH_glrlm_LowGrayLevelRunEmphasis | wavelet_LLH_glrlm_LowGrayLevelRunEmphasis |
| wavelet_LLH_glrlm_RunEntropy | wavelet_LLH_glrlm_RunEntropy |
| wavelet_LLH_glrlm_RunLengthNonUniformity | wavelet_LLH_glrlm_RunLengthNonUniformity |
| **wavelet_LLH_glrlm_RunLengthNonUniformityNormalized** | wavelet_LLH_glrlm_RunLengthNonUniformityNormalized |
| **wavelet_LLH_glrlm_RunPercentage** | wavelet_LLH_glrlm_RunPercentage |
| wavelet_LLH_glrlm_RunVariance | wavelet_LLH_glrlm_RunVariance |
| wavelet_LLH_glrlm_ShortRunEmphasis | wavelet_LLH_glrlm_ShortRunEmphasis |
| wavelet_LLH_glrlm_ShortRunHighGrayLevelEmphasis | wavelet_LLH_glrlm_ShortRunHighGrayLevelEmphasis |
| wavelet_LLH_glrlm_ShortRunLowGrayLevelEmphasis | wavelet_LLH_glrlm_ShortRunLowGrayLevelEmphasis |
| wavelet_LLH_glszm_GrayLevelNonUniformity | wavelet_LLH_glszm_GrayLevelNonUniformity |
| wavelet_LLH_glszm_GrayLevelNonUniformityNormalized | wavelet_LLH_glszm_GrayLevelNonUniformityNormalized |
| wavelet_LLH_glszm_GrayLevelVariance | wavelet_LLH_glszm_GrayLevelVariance |
| wavelet_LLH_glszm_HighGrayLevelZoneEmphasis | wavelet_LLH_glszm_HighGrayLevelZoneEmphasis |
| wavelet_LLH_glszm_LargeAreaEmphasis | wavelet_LLH_glszm_LargeAreaEmphasis |
| wavelet_LLH_glszm_LargeAreaHighGrayLevelEmphasis | wavelet_LLH_glszm_LargeAreaHighGrayLevelEmphasis |
| wavelet_LLH_glszm_LargeAreaLowGrayLevelEmphasis | wavelet_LLH_glszm_LargeAreaLowGrayLevelEmphasis |
| wavelet_LLH_glszm_LowGrayLevelZoneEmphasis | wavelet_LLH_glszm_LowGrayLevelZoneEmphasis |
| wavelet_LLH_glszm_SizeZoneNonUniformity | wavelet_LLH_glszm_SizeZoneNonUniformity |
| wavelet_LLH_glszm_SizeZoneNonUniformityNormalized | wavelet_LLH_glszm_SizeZoneNonUniformityNormalized |
| **wavelet_LLH_glszm_SmallAreaEmphasis** | **wavelet_LLH_glszm_SmallAreaEmphasis** |
| wavelet_LLH_glszm_SmallAreaHighGrayLevelEmphasis | wavelet_LLH_glszm_SmallAreaHighGrayLevelEmphasis |
| wavelet_LLH_glszm_SmallAreaLowGrayLevelEmphasis | wavelet_LLH_glszm_SmallAreaLowGrayLevelEmphasis |
| wavelet_LLH_glszm_ZoneEntropy | wavelet_LLH_glszm_ZoneEntropy |
| **wavelet_LLH_glszm_ZonePercentage** | **wavelet_LLH_glszm_ZonePercentage** |
| wavelet_LLH_glszm_ZoneVariance | wavelet_LLH_glszm_ZoneVariance |
| wavelet_LLH_ngtdm_Busyness | wavelet_LLH_ngtdm_Busyness |
| **wavelet_LLH_ngtdm_Coarseness** | **wavelet_LLH_ngtdm_Coarseness** |
| **wavelet_LLH_ngtdm_Complexity** | wavelet_LLH_ngtdm_Complexity |
| **wavelet_LLH_ngtdm_Contrast** | **wavelet_LLH_ngtdm_Contrast** |
| **wavelet_LLH_ngtdm_Strength** | wavelet_LLH_ngtdm_Strength |
| wavelet_LLL_glcm_Autocorrelation | wavelet_LLL_glcm_Autocorrelation |
| wavelet_LLL_glcm_ClusterProminence | wavelet_LLL_glcm_ClusterProminence |
| wavelet_LLL_glcm_ClusterShade | wavelet_LLL_glcm_ClusterShade |
| wavelet_LLL_glcm_ClusterTendency | wavelet_LLL_glcm_ClusterTendency |
| wavelet_LLL_glcm_Contrast | wavelet_LLL_glcm_Contrast |
| wavelet_LLL_glcm_Correlation | wavelet_LLL_glcm_Correlation |
| wavelet_LLL_glcm_DifferenceAverage | wavelet_LLL_glcm_DifferenceAverage |
| wavelet_LLL_glcm_DifferenceEntropy | wavelet_LLL_glcm_DifferenceEntropy |
| wavelet_LLL_glcm_DifferenceVariance | wavelet_LLL_glcm_DifferenceVariance |
| wavelet_LLL_glcm_Id | wavelet_LLL_glcm_Id |
| wavelet_LLL_glcm_Idm | wavelet_LLL_glcm_Idm |
| **wavelet_LLL_glcm_Idmn** | wavelet_LLL_glcm_Idmn |
| **wavelet_LLL_glcm_Idn** | wavelet_LLL_glcm_Idn |
| wavelet_LLL_glcm_Imc1 | wavelet_LLL_glcm_Imc1 |
| wavelet_LLL_glcm_Imc2 | wavelet_LLL_glcm_Imc2 |
| wavelet_LLL_glcm_InverseVariance | wavelet_LLL_glcm_InverseVariance |
| wavelet_LLL_glcm_JointAverage | wavelet_LLL_glcm_JointAverage |
| wavelet_LLL_glcm_JointEnergy | wavelet_LLL_glcm_JointEnergy |
| wavelet_LLL_glcm_JointEntropy | wavelet_LLL_glcm_JointEntropy |
| wavelet_LLL_glcm_MaximumProbability | wavelet_LLL_glcm_MaximumProbability |
| wavelet_LLL_glcm_SumAverage | wavelet_LLL_glcm_SumAverage |
| wavelet_LLL_glcm_SumEntropy | wavelet_LLL_glcm_SumEntropy |
| wavelet_LLL_glcm_SumSquares | wavelet_LLL_glcm_SumSquares |
| wavelet_LLL_gldm_DependenceEntropy | wavelet_LLL_gldm_DependenceEntropy |
| wavelet_LLL_gldm_DependenceNonUniformity | wavelet_LLL_gldm_DependenceNonUniformity |
| wavelet_LLL_gldm_DependenceNonUniformityNormalized | wavelet_LLL_gldm_DependenceNonUniformityNormalized |
| wavelet_LLL_gldm_DependenceVariance | wavelet_LLL_gldm_DependenceVariance |
| wavelet_LLL_gldm_GrayLevelNonUniformity | wavelet_LLL_gldm_GrayLevelNonUniformity |
| wavelet_LLL_gldm_GrayLevelVariance | wavelet_LLL_gldm_GrayLevelVariance |
| wavelet_LLL_gldm_HighGrayLevelEmphasis | wavelet_LLL_gldm_HighGrayLevelEmphasis |
| **wavelet_LLL_gldm_LargeDependenceEmphasis** | wavelet_LLL_gldm_LargeDependenceEmphasis |
| wavelet_LLL_gldm_LargeDependenceHighGrayLevelEmphasis | wavelet_LLL_gldm_LargeDependenceHighGrayLevelEmphasis |
| wavelet_LLL_gldm_LargeDependenceLowGrayLevelEmphasis | wavelet_LLL_gldm_LargeDependenceLowGrayLevelEmphasis |
| wavelet_LLL_gldm_LowGrayLevelEmphasis | wavelet_LLL_gldm_LowGrayLevelEmphasis |
| **wavelet_LLL_gldm_SmallDependenceEmphasis** | wavelet_LLL_gldm_SmallDependenceEmphasis |
| wavelet_LLL_gldm_SmallDependenceHighGrayLevelEmphasis | wavelet_LLL_gldm_SmallDependenceHighGrayLevelEmphasis |
| **wavelet_LLL_gldm_SmallDependenceLowGrayLevelEmphasis** | wavelet_LLL_gldm_SmallDependenceLowGrayLevelEmphasis |
| wavelet_LLL_glrlm_GrayLevelNonUniformity | wavelet_LLL_glrlm_GrayLevelNonUniformity |
| wavelet_LLL_glrlm_GrayLevelNonUniformityNormalized | wavelet_LLL_glrlm_GrayLevelNonUniformityNormalized |
| wavelet_LLL_glrlm_GrayLevelVariance | wavelet_LLL_glrlm_GrayLevelVariance |
| wavelet_LLL_glrlm_HighGrayLevelRunEmphasis | wavelet_LLL_glrlm_HighGrayLevelRunEmphasis |
| **wavelet_LLL_glrlm_LongRunEmphasis** | wavelet_LLL_glrlm_LongRunEmphasis |
| wavelet_LLL_glrlm_LongRunHighGrayLevelEmphasis | wavelet_LLL_glrlm_LongRunHighGrayLevelEmphasis |
| wavelet_LLL_glrlm_LongRunLowGrayLevelEmphasis | wavelet_LLL_glrlm_LongRunLowGrayLevelEmphasis |
| wavelet_LLL_glrlm_LowGrayLevelRunEmphasis | wavelet_LLL_glrlm_LowGrayLevelRunEmphasis |
| **wavelet_LLL_glrlm_RunEntropy** | wavelet_LLL_glrlm_RunEntropy |
| wavelet_LLL_glrlm_RunLengthNonUniformity | wavelet_LLL_glrlm_RunLengthNonUniformity |
| **wavelet_LLL_glrlm_RunLengthNonUniformityNormalized** | wavelet_LLL_glrlm_RunLengthNonUniformityNormalized |
| **wavelet_LLL_glrlm_RunPercentage** | wavelet_LLL_glrlm_RunPercentage |
| **wavelet_LLL_glrlm_RunVariance** | wavelet_LLL_glrlm_RunVariance |
| **wavelet_LLL_glrlm_ShortRunEmphasis** | wavelet_LLL_glrlm_ShortRunEmphasis |
| wavelet_LLL_glrlm_ShortRunHighGrayLevelEmphasis | wavelet_LLL_glrlm_ShortRunHighGrayLevelEmphasis |
| **wavelet_LLL_glrlm_ShortRunLowGrayLevelEmphasis** | wavelet_LLL_glrlm_ShortRunLowGrayLevelEmphasis |
| wavelet_LLL_glszm_GrayLevelNonUniformity | wavelet_LLL_glszm_GrayLevelNonUniformity |
| wavelet_LLL_glszm_GrayLevelNonUniformityNormalized | wavelet_LLL_glszm_GrayLevelNonUniformityNormalized |
| wavelet_LLL_glszm_GrayLevelVariance | wavelet_LLL_glszm_GrayLevelVariance |
| wavelet_LLL_glszm_HighGrayLevelZoneEmphasis | wavelet_LLL_glszm_HighGrayLevelZoneEmphasis |
| wavelet_LLL_glszm_LargeAreaEmphasis | wavelet_LLL_glszm_LargeAreaEmphasis |
| wavelet_LLL_glszm_LargeAreaHighGrayLevelEmphasis | wavelet_LLL_glszm_LargeAreaHighGrayLevelEmphasis |
| wavelet_LLL_glszm_LargeAreaLowGrayLevelEmphasis | wavelet_LLL_glszm_LargeAreaLowGrayLevelEmphasis |
| wavelet_LLL_glszm_LowGrayLevelZoneEmphasis | wavelet_LLL_glszm_LowGrayLevelZoneEmphasis |
| wavelet_LLL_glszm_SizeZoneNonUniformity | wavelet_LLL_glszm_SizeZoneNonUniformity |
| wavelet_LLL_glszm_SizeZoneNonUniformityNormalized | wavelet_LLL_glszm_SizeZoneNonUniformityNormalized |
| wavelet_LLL_glszm_SmallAreaEmphasis | wavelet_LLL_glszm_SmallAreaEmphasis |
| wavelet_LLL_glszm_SmallAreaHighGrayLevelEmphasis | wavelet_LLL_glszm_SmallAreaHighGrayLevelEmphasis |
| wavelet_LLL_glszm_SmallAreaLowGrayLevelEmphasis | wavelet_LLL_glszm_SmallAreaLowGrayLevelEmphasis |
| **wavelet_LLL_glszm_ZoneEntropy** | wavelet_LLL_glszm_ZoneEntropy |
| **wavelet_LLL_glszm_ZonePercentage** | wavelet_LLL_glszm_ZonePercentage |
| wavelet_LLL_glszm_ZoneVariance | wavelet_LLL_glszm_ZoneVariance |
| wavelet_LLL_ngtdm_Busyness | wavelet_LLL_ngtdm_Busyness |
| **wavelet_LLL_ngtdm_Coarseness** | **wavelet_LLL_ngtdm_Coarseness** |
| **wavelet_LLL_ngtdm_Complexity** | wavelet_LLL_ngtdm_Complexity |
| **wavelet_LLL_ngtdm_Contrast** | **wavelet_LLL_ngtdm_Contrast** |
| wavelet_LLL_ngtdm_Strength | wavelet_LLL_ngtdm_Strength |
|  |  |
|  |  |
